# Hematopoietic loss of KDM6A impairs cardiac recovery in heart failure via epigenetic reprogramming of myeloid cells

**DOI:** 10.1038/s44161-026-00887-3

**Published:** 2026-11-03

**Authors:** Emmanouil G. Solomonidis, Dennis Hecker, Mariana Shumliakivska, Julian Leberzammer, Ariane Fischer, Minh-Thuy Katschke, Melanie Bendel, Lara Korth, Bianca Schuhmacher, Laura Rumpf, Simone-Franziska Glaser, Katja Schmitz, Guillermo Luxán, Silke Frenz, Penelope Pennoyer, Annett Wilken-Schmitz, Tina Rasper, Sinan Zimmermann, Franziska Ganß, Evelyn Ullrich, David John, Stefan Guenther, Katalin Pálfi, Lisa M. Weiss, Matthias S. Leisegang, Ralf P. Brandes, Moritz von Scheidt, David M. Leistner, Andreas M. Zeiher, Wesley T. Abplanalp, Mario Looso, Marcel H. Schulz, Stefanie Dimmeler, Sebastian Cremer

**Affiliations:** 1 Institute of Cardiovascular Regeneration, Center of Molecular Medicine, Goethe University Frankfurt, Frankfurt am Main, Germany; 2 Cardiopulmonary Institute, Goethe University Frankfurt, Frankfurt am Main, Germany; 3 German Center for Cardiovascular Research (DZHK), Partner site Rhine-Main, Frankfurt am Main, Germany; 4 Institute for Computational Genomic Medicine, Goethe University Frankfurt, Frankfurt am Main, Germany; 5 Department of Medicine, Cardiology, University Hospital Frankfurt, Frankfurt am Main, Germany; 6 Experimental Immunology, Department for Children and Adolescent Medicine, University Hospital Frankfurt, Goethe University Frankfurt, Frankfurt am Main, Germany; 7 Max Planck Institute for Heart and Lung Research, Bad Nauheim, Germany; 8 Institute of Cardiovascular Physiology, Goethe University, Frankfurt am Main, Germany; 9 Department of Cardiology, TUM University Hospital German Heart Center, School of Medicine and Health, Technical University of Munich, Munich, Germany; 10 Deutsches Zentrum für Herz und Kreislaufforschung (DZHK), Partner Site Munich Heart Alliance, Munich, Germany

**Keywords:** Heart failure, Translational research

## Abstract

Clonal hematopoiesis (CH), the expansion of blood cell clones carrying acquired mutations, is an independent risk factor for cardiovascular disease. Although mutations in *DNMT3A* and *TET2* are well studied, the cardiovascular consequences of *KDM6A* mutations remain unclear. KDM6A is an X-linked histone demethylase that is commonly mutated in patients with heart failure (HF). Here we performed multiomics profiling and functional characterization of mouse models and patient-derived data to show that hematopoietic KDM6A loss impairs cardiac recovery after myocardial infarction (MI). KDM6A deficiency increases myeloid cell recruitment to the injured heart and reprograms monocytes, macrophages and neutrophils toward inflammatory, migratory and glycolytic states. Patients with HF and KDM6A-driven CH show similar pro-inflammatory monocyte signatures. KDM6A-silenced macrophages promote cardiomyocyte hypertrophy and cardiac fibroblast activation. Notably, IL-1β blockade after MI rescued the adverse cardiac phenotype of hematopoietic KDM6A loss. These findings identify KDM6A-driven CH as a driver of immune dysregulation and IL-1β signaling as a potential therapeutic target in CH-associated HF.

## Abstract (web summary)

Solomonidis et al. reveal that hematopoietic KDM6A-driven clonal hematopoiesis worsens post-MI cardiac recovery by promoting inflammatory myeloid reprogramming. IL-1β blockade reverses these adverse effects, highlighting a potential therapeutic strategy.

## Main

CH is increasingly recognized as an independent risk factor for cardiovascular disease (CVD)^1^. Although mutations in commonly studied genes such as *DNMT3A* and *TET2* have been robustly linked to worse cardiovascular outcomes, many less frequently mutated genes remain poorly characterized. Recent large-scale analyses continue to identify novel and understudied CH-associated mutations that may promote disease through distinct mechanisms, emphasizing the potential for personalized medicine strategies tailored to specific genetic profiles^2^.

Among these understudied mutated genes is lysine demethylase 6A (KDM6A, also known as UTX), an X-linked histone demethylase^3^. The primary role of KDM6A is demethylation of H3 lysine 27 dimethylation and trimethylation (H3K27me2/H3K27me3), marks commonly associated with transcriptional repression^4^. UTY is the Y chromosome homolog of KDM6A but displays weaker demethylase activity^5^. Recently, *KDM6A* emerged as one of the most commonly mutated genes among patients with HF^6^. However, the mechanistic contribution of *KDM6A* mutations to cardiovascular pathology remains unexplored.

In the present study, we employed a comprehensive multiomics approach in mouse models and patient-derived data to determine how hematopoietic loss of KDM6A function influences inflammation and cardiac dysfunction after ischemic injury. We reveal that hematopoietic KDM6A loss profoundly remodels chromatin and transcriptional programs in immune cells, resulting in augmented inflammatory responses and worsening cardiac function after MI. Notably, we show that IL-1β inhibition rescues the detrimental effects of hematopoietic KDM6A deficiency. These findings offer critical insights into CH-driven cardiovascular pathology and position patients with HF harboring KDM6A mutations, as well as patients with CVD with pro-inflammatory CH more broadly, as promising candidates for personalized therapeutic interventions targeting inflammation.

## Results

### Hematopoietic loss of KDM6A impairs recovery of cardiac function after MI

**Fig. 1:**
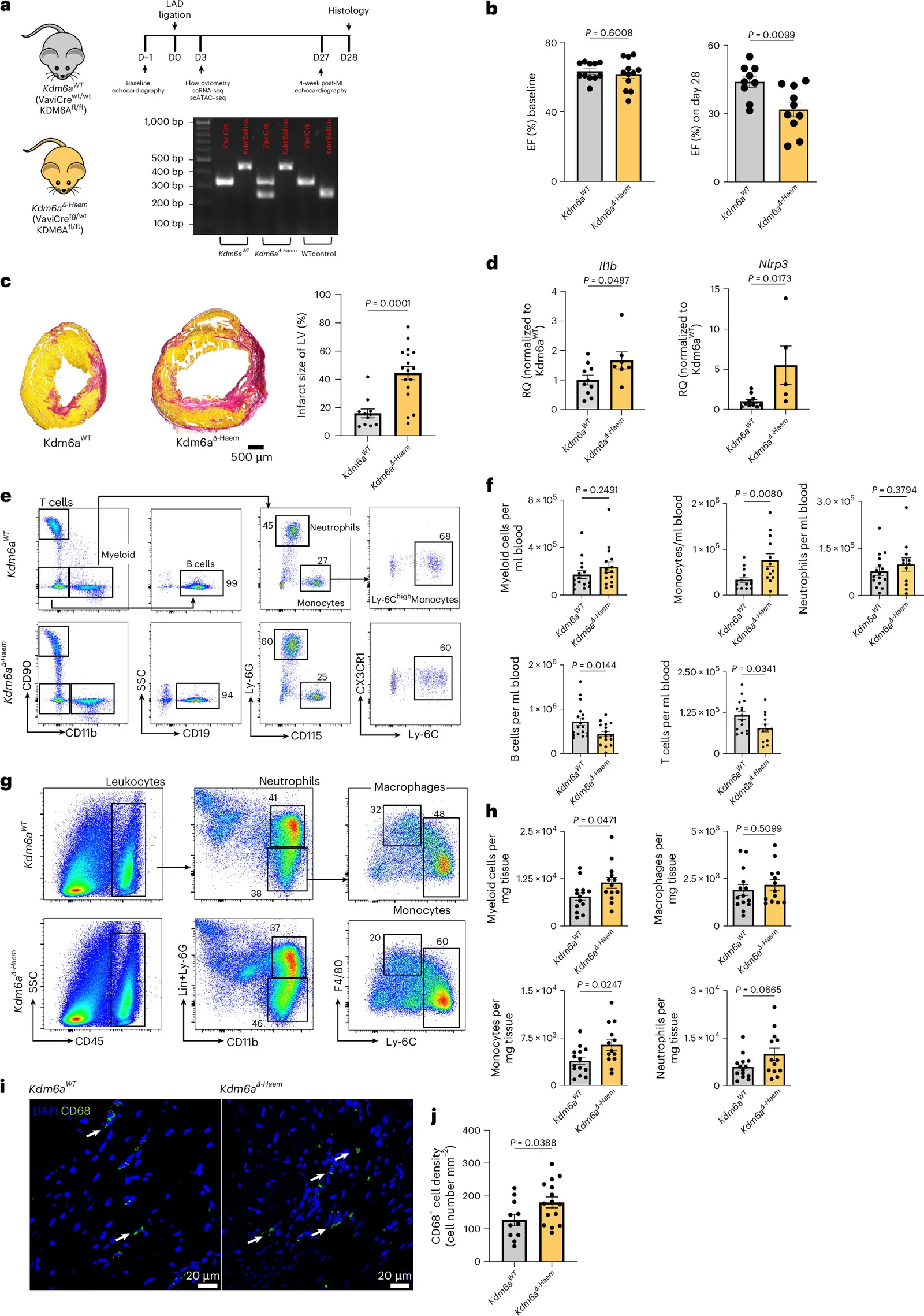
Hematopoietic loss of KDM6A impacts cardiac recovery while displaying pro-inflammatory features. **a**, Experimental design of mouse studies with hematopoietic-specific KDM6A knockout. Agarose gel electrophoresis showing representative genotypes of *Kdm6a*^WT^ (VaviCre^wt/wt^KDM6A^fl/fl^), *Kdm6a*^*Δ-Haem*^ (VaviCre^tg/wt^KDM6A^fl/fl^) and WT mice (VaviCre genotype: internal positive control band 324 bp, Cre transgene band 236 bp; KDM6A flox genotype: homozygous flox band 430 bp, WT band 249 bp). **b**, Baseline cardiac EF (*Kdm6a*^WT^ *n* = 11 versus *Kdm6a*^*Δ-Haem*^ *n* = 12, *P* = 0.601) and 28 days after MI (*Kdm6a*^WT^ *n* = 9 versus *Kdm6a*^*Δ-Haem*^ *n* = 10, *P* = 0.001). **c**, Representative images of picrosirius red cardiac tissue staining 28 days after MI in *Kdm6a*^WT^ and *Kdm6a*^*Δ-Haem*^ mice (scale, 500 μm). Quantification of scar size (*Kdm6a*^WT^ *n* = 11 versus *Kdm6a*^*Δ-Haem*^ *n* = 17, *P* = 0.0001). **d**, qPCR data from RNA extracted from cardiac tissue 28 days after MI for *Il1b* (*Kdm6a*^WT^ *n* = 10 versus *Kdm6a*^*Δ-Haem*^ *n* = 7, *P* = 0.04) and *Nlrp3* (*Kdm6a*^WT^ *n* = 10 versus *Kdm6a*^*Δ-Haem*^ *n* = 5, *P* = 0.012). **e**, Flow cytometry gating of blood leukocytes 3 days after MI; numbers refer to percentage of parent gate. **f**, Flow cytometric analysis of blood leukocytes 3 days after MI; CD11b^+^ myeloid cells (*Kdm6a*^WT^ *n* = 15 versus *Kdm6a*^*Δ-Haem*^ *n* = 15, *P* = 0.249), monocytes (*Kdm6a*^WT^ *n* = 14 versus *Kdm6a*^*Δ-Haem*^ *n* = 13, *P* = 0.008), B cells (*Kdm6a*^WT^ *n* = 18 versus *Kdm6a*^*Δ-Haem*^ *n* = 16, *P* = 0.014) and T cells (*Kdm6a*^WT^ *n* = 15 versus *Kdm6a*^*Δ-Haem*^ *n* = 13, *P* = 0.034). **g**, Flow cytometry gating of cardiac leukocytes 3 days after MI in *Kdm6a*^WT^ and *Kdm6a*^*Δ-Haem*^ mice; numbers refer to percentage of parent gate. **h**, Flow cytometric analysis of cardiac leukocytes 3 days after MI; CD11b^+^ myeloid cells (*Kdm6a*^WT^ *n* = 14 versus *Kdm6a*^*Δ-Haem*^ *n* = 13, *P* = 0.047), macrophages (*Kdm6a*^WT^ *n* = 14 versus *Kdm6a*^*Δ-Haem*^ *n* = 13, *P* = 0.501), monocytes (*Kdm6a*^WT^ *n* = 14 versus *Kdm6a*^*Δ-Haem*^ *n* = 13, *P* = 0.025) and neutrophils (*Kdm6a*^WT^ *n* = 14 versus *Kdm6a*^*Δ-Haem*^ *n* = 13, *P* = 0.066). **i**, Representative images of immunostaining of CD68^+^ macrophages in cardiac tissue 28 days after MI in *Kdm6a*^WT^ and *Kdm6a*^*Δ-Haem*^ mice. White arrows point to CD68^+^ macrophages. Scale bars, 20 μm. **j**, Quantification of CD68^+^ macrophages 28 days after MI in *Kdm6a*^WT^ and *Kdm6a*^*Δ-Haem*^ mice (*Kdm6a*^WT^ *n* = 11 versus *Kdm6a*^*Δ-Haem*^ *n* = 15, *P* = 0.039). Data are represented as mean ± s.e.m.; *P* values were calculated using two-tailed unpaired *t*-tests. D, day; LV, left ventricle; RQ, relative quantification. Credit: mouse icon in **a** adapted from Openclipart under a Creative Commons licence CC0 1.0. Source data

**Extended Data Fig. 1:**
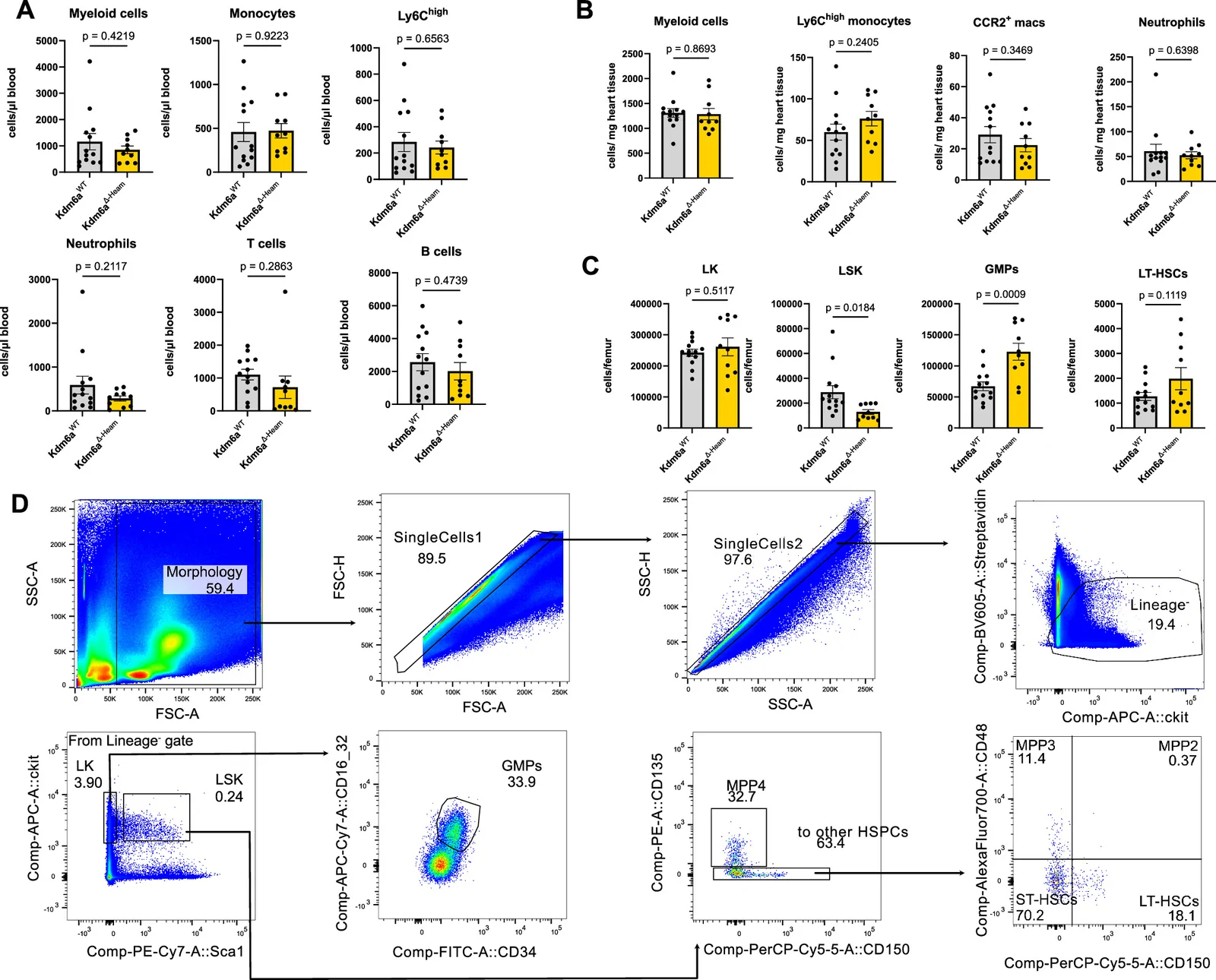
Baseline characterisation of peripheral leukocytes, cardiac myeloid cells and haematopoietic progenitors in Kdm6a^Δ-Haem^ mice. (**A**) Peripheral leukocytes analysis with flow cytometry (**B**) Cardiac myeloid cell analysis in the uninjured Kdm6a^Δ-haem^ with flow cytometry (**C**) Haematopoietic progenitor composition in the bone marrow of uninjured Kdm6a^Δ-haem^. (**D**) Example of bone marrow flow cytometry gating. Statistical analysis was done with an unpaired t-test. Data points in graphs represent individual biological replicates and presented as mean ± SEM. Source data

**Extended Data Fig. 2:**
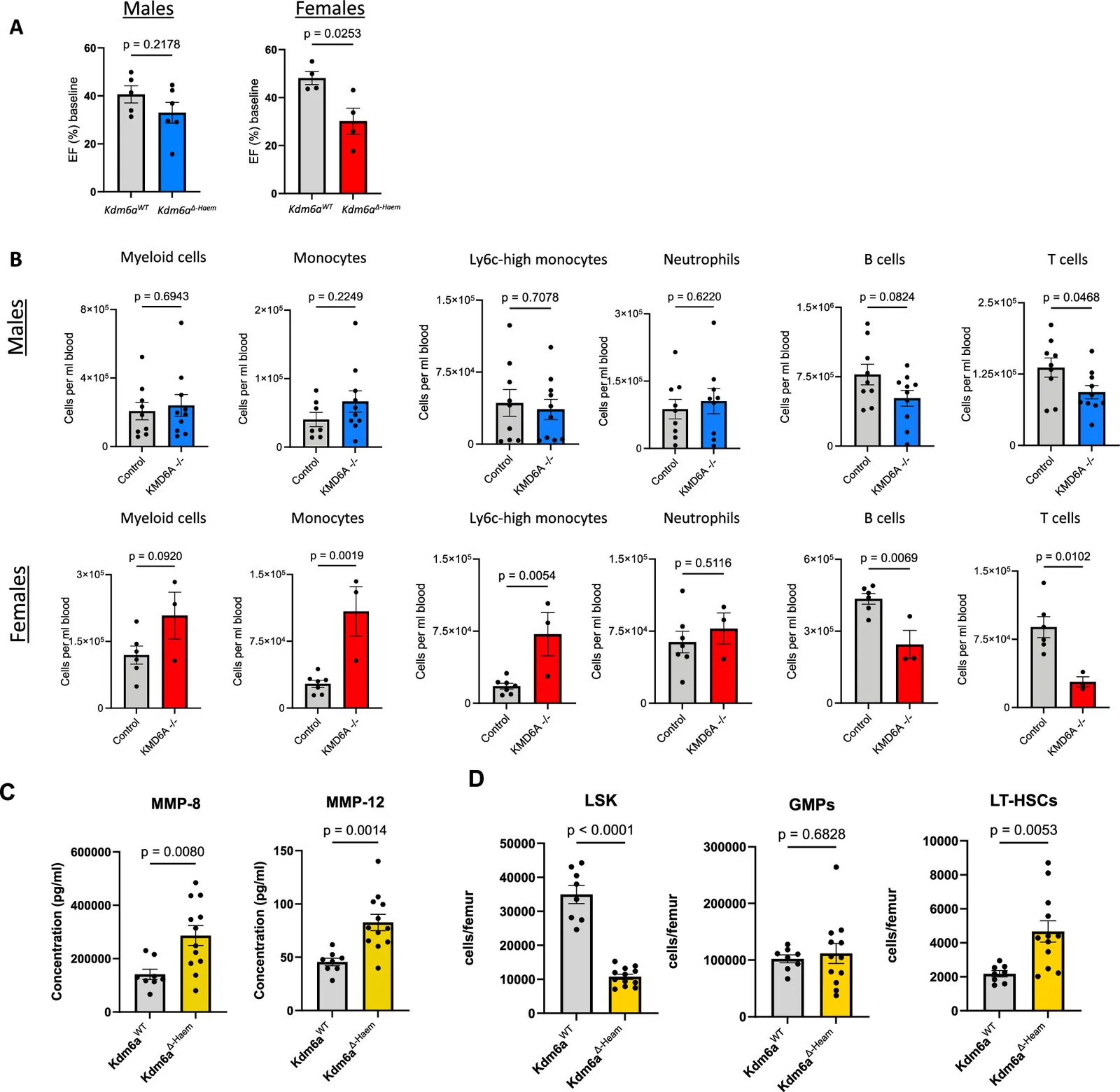
Echocardiography and flow cytometric analysis of Kdm6a^Δ-Haem^ mice. (**A**) Cardiac ejection fraction (EF) 28 days post-myocardial infarction (MI) for male (*Kdm6a*^*WT*^ n = 5 vs *Kdm6a*^*Δ-Haem*^ n = 6, p = 0.2178) and female *Kdm6a*^*Δ-Haem*^ mice (*Kdm6a*^*WT*^ n = 4 vs *Kdm6a*^*Δ-Haem*^ n = 4, p = 0.0253) (**B**) Flow cytometric analysis of blood leukocytes of male and female *Kdm6a*^*Δ-Haem*^ mice at 3 days post-MI. Data are represented as mean ± SEM, p-values were calculated using two-tailed unpaired t-tests. (**C**) ELISA from peripheral plasma at 3 days post-myocardial infarction (MI) in Kdm6a^Δ-Haem^ mice (**D**) Haematopoietic progenitor composition in the bone marrow of Kdm6a^Δ-haem^ mice at 3 days post-MI. Statistical analysis was done with a two-tail unpaired t-test. Data points in graphs represent individual biological replicates and presented as mean ± SEM. Source data

To investigate how hematopoietic loss of KDM6A affects HF, we crossed *VaviCre* mice with *Kdm6a*^*fl*/*fl*^ mice, generating mice with hematopoietic-specific Kdm6a knockout (*Kdm6a*^*Δ-Haem*^; Fig. 1a). Baseline ejection fraction (EF) showed no differences between groups (Fig. 1b). Baseline *Kdm6a*^*Δ-Haem*^ mice did not show increased mature myeloid cell abundance in peripheral blood (Extended Data Fig. 1a). Similarly, baseline cardiac immune profiling showed no increase in total cardiac myeloid cells, neutrophils, Ly6C^high^ monocytes or CCR2^+^ macrophages (Extended Data Fig. 1b). By contrast, bone marrow profiling revealed that KDM6A loss alters the baseline hematopoietic stem and progenitor compartment with increased granulocyte−monocyte progenitors (GMPs), whereas Lin^−^Sca1^−^cKit^+^ (LK) cells were unchanged, Lin^−^Sca1^+^cKit^+^ (LSK) cells were reduced and long-term hematopoietic stem cells (LT-HSCs) showed a trend toward increase (Extended Data Fig. 1c,d). After MI induced by permanent occlusion of the left anterior descending (LAD) coronary artery, *Kdm6a*^*Δ-Haem*^ mice displayed reduced EF compared to *Kdm6a*^WT^ mice at day 28 after MI (Fig. 1b). Both male and female *Kdm6a*^*Δ-Haem*^ mice exhibited impaired EF compared to *Kdm6a*^WT^ controls at day 28 after MI; however, the phenotype was more pronounced in females (Extended Data Fig. 2a). Additionally, *Kdm6a*^*Δ-Haem*^ hearts exhibited increased left ventricular scar size (Fig. 1c) and increased expression of pro-inflammatory markers *Il1b* and *Nlrp3* at day 28 after MI (Fig. 1d). Analysis of the circulating leukocyte composition in peripheral blood revealed monocytosis at day 3 after MI, the peak of inflammatory activity after MI, specifically in female *Kdm6a*^*Δ-Haem*^ mice, compared to controls (Fig. 1e,f and Extended Data Fig. 2b). T lymphocytes were reduced in both sexes at day 3 after MI, whereas B cells were significantly decreased in females, with a similar trend observed in males (Fig. 1e,f and Extended Data Fig. 2b). *Kdm6a*^*Δ-Haem*^ mice also showed increased circulating matrix metalloproteinase-8 (MMP-8) and matrix metalloproteinase-12 (MMP-12) in *Kdm6a*^*Δ-Haem*^ mice (Extended Data Fig. 2c), linked to adverse post-MI remodeling^7,8^, suggesting that KDM6A loss amplifies the systemic inflammatory/remodeling response to MI.

Cardiac immune cell profiling at day 3 after MI revealed increased infiltration of myeloid cells, predominantly monocytes, into the ischemic myocardium (Fig. 1g,h). GMP numbers were no longer increased, whereas LSK cells were reduced and LT-HSCs were increased (Extended Data Fig. 2d). Notably, GMP numbers appeared to plateau, suggesting that the increased circulating monocytes and enhanced myocardial myeloid recruitment at day 3 are not explained by further numerical expansion of the GMP pool at this timepoint.

**Extended Data Fig. 3:**
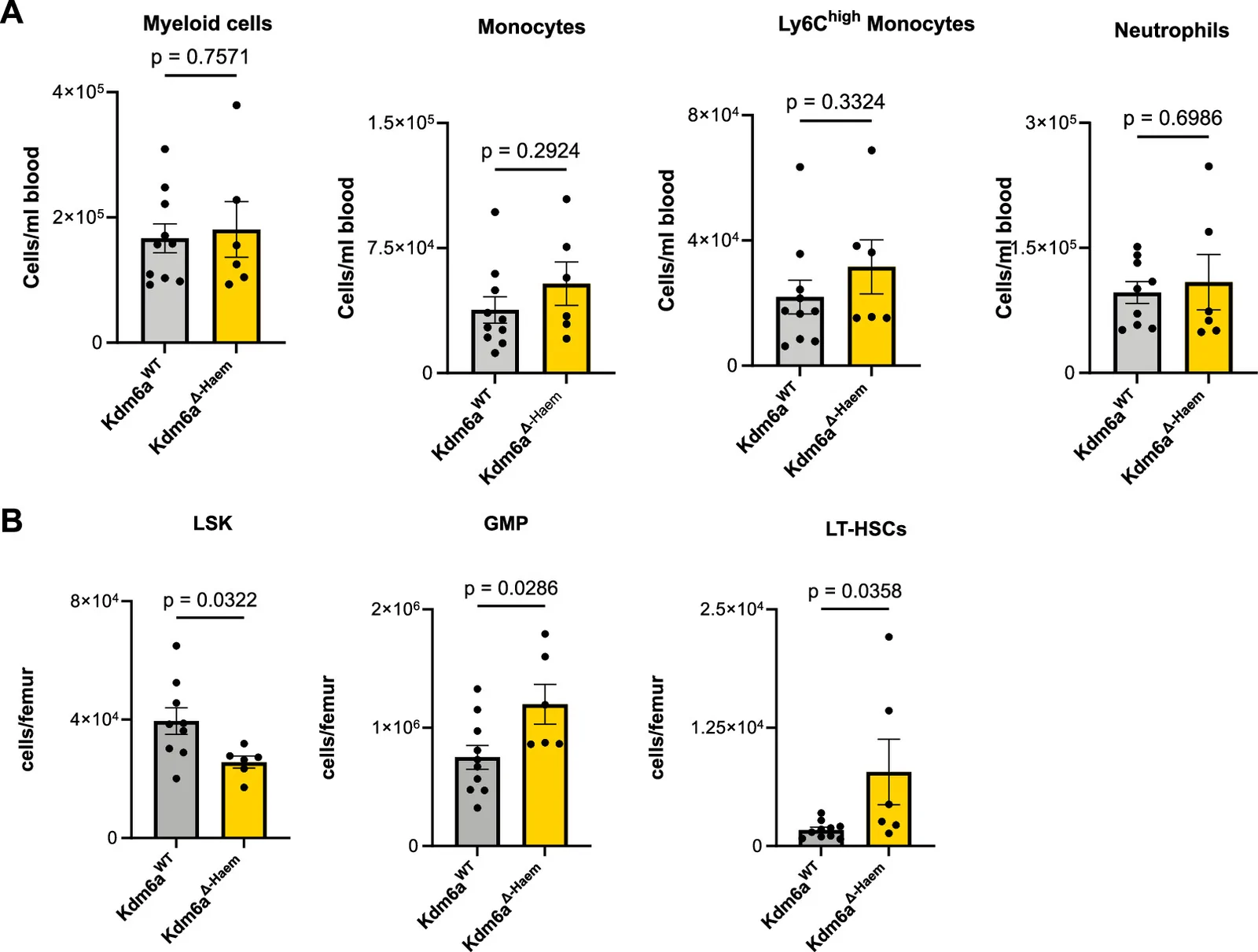
Peripheral leukocyte profiling and haematopoietic composition analysis in Kdm6a^Δ-Haem^ mice at 28 days post-MI. (**A**) Peripheral leukocytes analysis with flow cytometry (**B**) Haematopoietic progenitor composition in the bone marrow of Kdm6a^Δ-haem^ mice at 28 days post-MI with flow cytometry. Statistical analysis was done with a two-tail unpaired t-test. Data points in graphs represent individual biological replicates and presented as mean ± SEM. Source data

At day 28 after MI, circulating myeloid cell numbers did not differ between *Kdm6a*^*Δ-Haem*^ and *Kdm6a*^WT^ mice, including total myeloid cells, monocytes and neutrophils (Extended Data Fig. 3a). However, this normalization in peripheral blood did not mean complete resolution of the KDM6A-loss phenotype as histological staining confirmed an increased number of CD68^+^ macrophages in cardiac tissue (Fig. 1i,j). In parallel, bone marrow abnormalities also persisted at this later remodeling stage, with reduced LSK cells, increased LT-HSCs and increased GMPs in *Kdm6a*^*Δ-Haem*^ mice (Extended Data Fig. 3b).

Together, these data indicate that hematopoietic KDM6A loss alters hematopoietic progenitor dynamics and enhances systemic inflammation, cardiac inflammation and myeloid cell recruitment into the myocardium, resulting in increased cardiac dysfunction after MI.

### KDM6A loss enhances inflammatory transcriptional and epigenetic activation in recruited macrophages after MI

As KDM6A acts primarily as a transcriptional activator via H3K27me2/3 demethylation^3^, we next investigated how hematopoietic KDM6A loss affects the chromatin accessibility and expression signature of hematopoietic cells after MI by performing single-cell assay for transposase-accessible chromatin using sequencing (scATAC−seq) and single-cell RNA sequencing (scRNA-seq) on CD45^+^ immune cells isolated from cardiac tissue of *Kdm6a*^*Δ-Haem*^ mice at 3 days after MI. We selected female mice due to their more pronounced impairment in EF after MI and increased systemic inflammation and to avoid potential compensatory effects from Y chromosome homologs.

**Extended Data Fig. 4:**
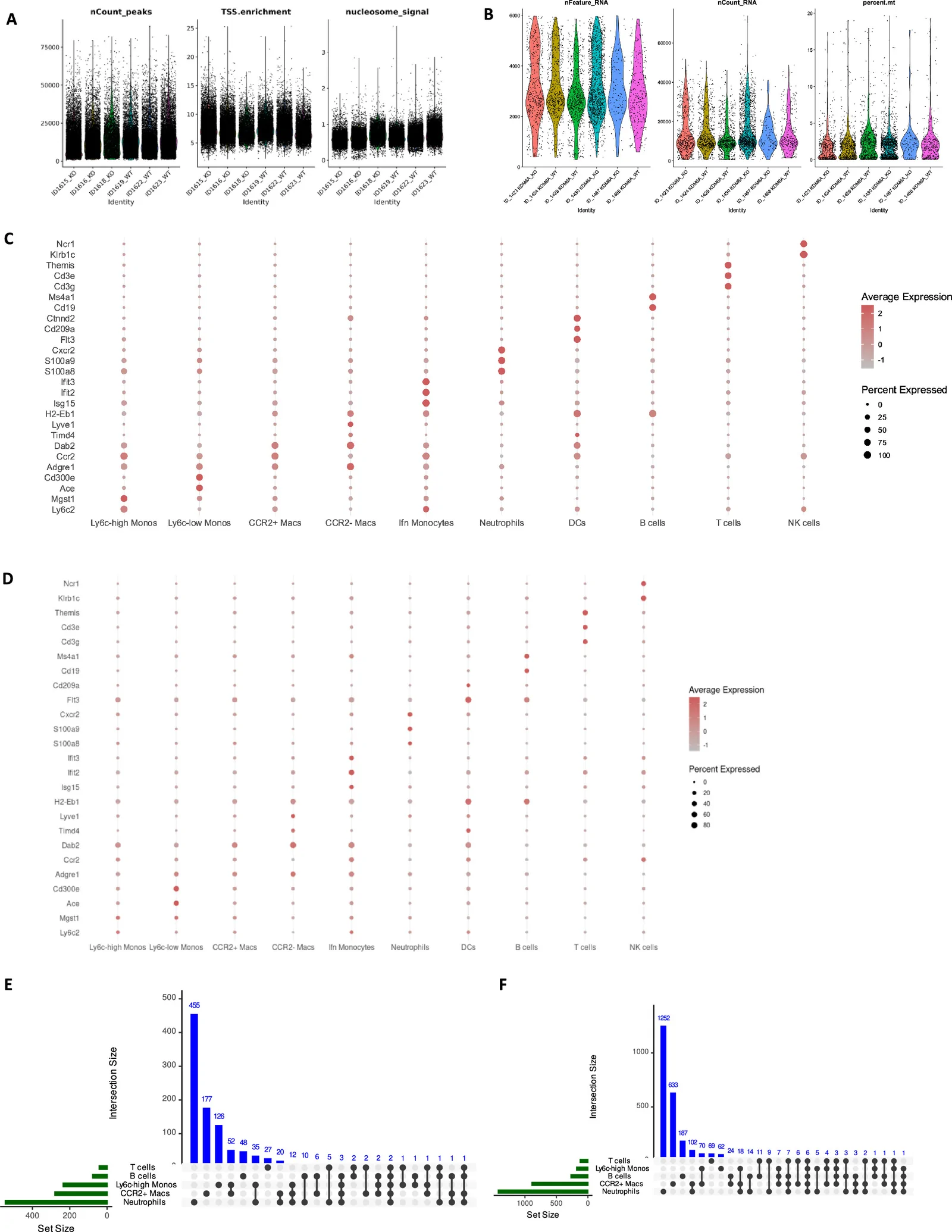
Quality control and annotation of mouse scATAC-seq and scRNA-seq data. (**A**) Quality control metrics for filtered scATAC-seq data (**B**) Quality control metrics for filtered scRNA-seq data (**C**) RNA expression of key markers used for annotation of leukocyte subsets in scRNA-seq data (**D**) Gene activity for markers used for scRNA-seq annotation, as assessed by the chromatin accessibility associated with the respective gene (**E**) Intersection plot per cell type of differentially expressed genes from scRNA-seq data (**F**) Intersection plot per cell type of differentially accessible peaks from scATAC-seq data. Source data

After quality control (Extended Data Fig. 4a,b), annotation of immune cell clusters was performed. Due to sparse data from scATAC−seq, we transferred cell type labels from non-paired scRNA-seq data, based on marker gene expression (Extended Data Fig. 4c). We crosschecked the successful annotation by checking the gene activity for markers used for scRNA-seq annotation, as assessed by the chromatin accessibility associated with the respective gene (Extended Data Fig. 4d).

**Fig. 2:**
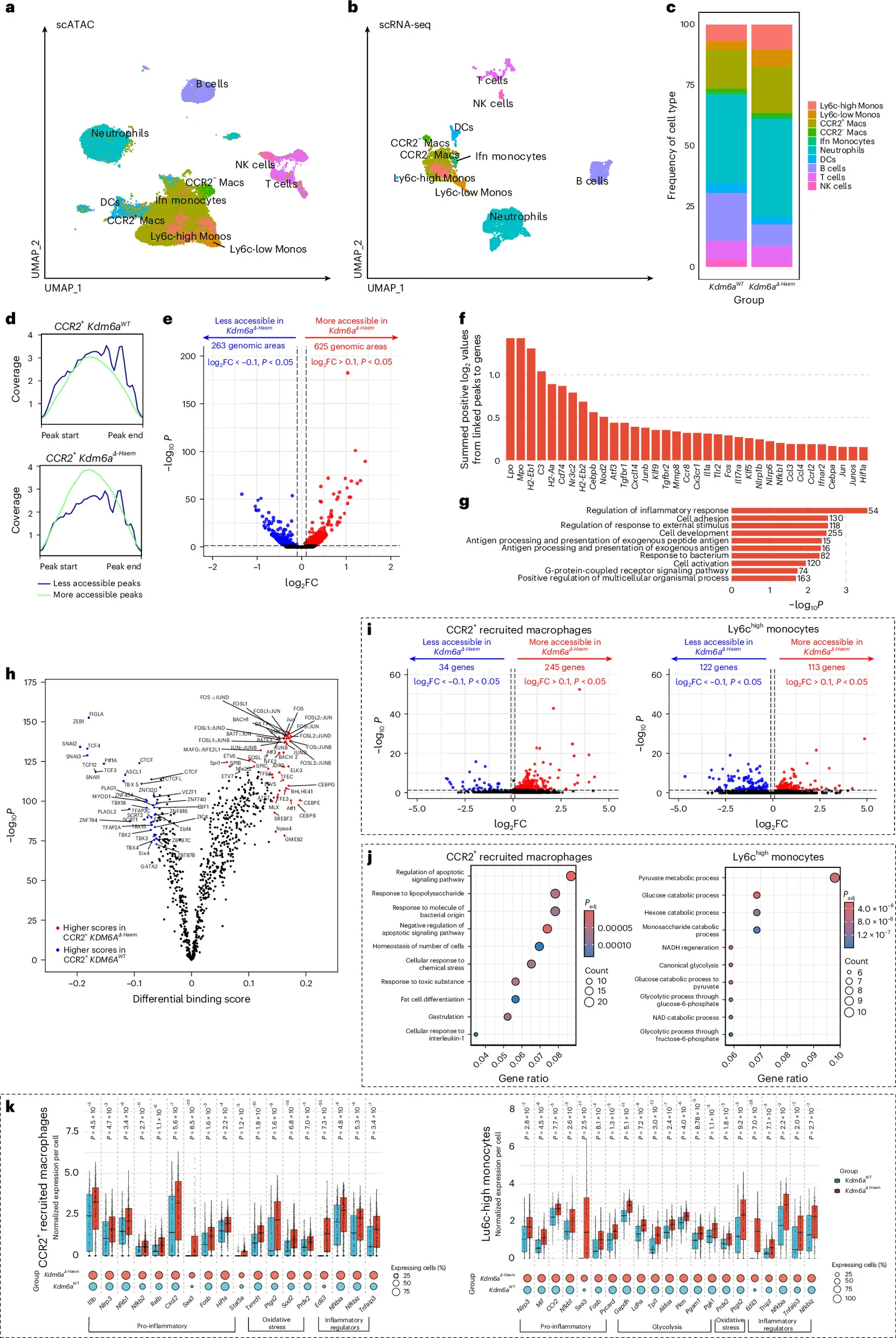
KDM6A loss enhances inflammatory transcriptional and epigenetic activation in recruited macrophages after MI. **a**, UMAP clustering of scATAC−seq data from cardiac CD45^+^ cells 3 days after MI in *Kdm6a*^WT^ and *Kdm6a*^*Δ-Haem*^ mice (*n* = 3 female mice per group). **b**, UMAP clustering of scRNA-seq data from cardiac CD45^+^ cells 3 days after MI in *Kdm6a*^WT^ and *Kdm6a*^*Δ-Haem*^ mice (*n* = 3 female mice per group). **c**, Cell type distribution in *Kdm6a*^WT^ and *Kdm6a*^*Δ-Haem*^ cell clusters from scRNA-seq data. **d**, ATAC−seq coverage plots showing average chromatin accessibility signal (average CPM) at genomic regions identified as differentially accessible peaks in CCR2^+^ macrophages from *Kdm6a*^WT^ (top) and *Kdm6a*^*Δ-Haem*^ (bottom) mice. **e**, Volcano plot of differentially accessible peaks in *Kdm6a*^WT^ and *Kdm6a*^*Δ-Haem*^ CCR2^+^ recruited macrophages (peak cutoff; 10% detection in either *Kdm6a*^WT^ or *Kdm6a*^*Δ-Haem*^ group). **f**, Summed positive log_2_ fold change values from differentially accessible chromatin peaks linked to genes in *Kdm6a*^*Δ-Haem*^ CCR2^+^ recruited macrophages. **g**, Top enriched Gene Ontology terms enriched from enhancers linked to genes in *Kdm6a*^*Δ-Haem*^ CCR2^+^ recruited macrophages. Numbers next to bars indicate the number of genes enriched in each Gene Ontology. **h**, Differential transcription factor binding activity predicted with the TOBIAS tool from chromatin accessibility data in CCR2^+^ macrophages from *Kdm6a*^*Δ-Haem*^ compared to *Kdm6a*^WT^ mice. **i**, Volcano plots of differentially expressed genes in CCR2^+^ recruited macrophages and Ly6c^high^ monocytes (gene cutoff; 10% expression in either *Kdm6a*^WT^ or *Kdm6a*^*Δ-Haem*^ group). **j**, Top enriched Gene Ontology terms enriched in differentially upregulated genes in *Kdm6a*^*Δ-Haem*^ CCR2^+^ macrophages and *Kdm6a*^*Δ-Haem*^ Ly6c^high^ monocytes. **k**, Normalized gene expression data from scRNA-seq per cell (top) and the percentage of expressing cells (bottom) for differentially upregulated genes in CCR2^+^ recruited macrophages (left) and Ly6c^high^ monocytes (right) from *Kdm6a*^*Δ-Haem*^ versus *Kdm6a*^WT^ mice. Differentially expressed genes were identified using the two-tailed Wilcoxon rank-sum test with Bonferroni correction for multiple comparisons. Box plots show the median (center line) and the 25th and 75th percentiles (lower and upper box boundaries). Whiskers extend to the lowest and highest observations within 1.5× the interquartile range below and above the box. Data points represent individual cells derived from *n* = 3 mice per group (*Kdm6a*^WT^ versus *Kdm6a*^*Δ-Haem*^). DC, dendritic cell; FC, fold change; Macs, macrophages; Monos, monocytes; NK, natural killer; *P*_adj_, adjusted *P* value.

Clustering identified distinct immune subsets, including CCR2^+^ recruited macrophages, Ly6c^high^ monocytes, Ly6c^low^ monocytes, resident CCR2^−^ macrophages, interferon (IFN)-activated monocytes, dendritic cells, neutrophils, T cells and B cells (Fig. 2a,b). Consistent with our flow cytometry data, *Kdm6a*^*Δ-Haem*^ mice displayed increased frequency of myeloid cells, particularly CCR2^+^ macrophages and their Ly6c^high^ monocyte precursors (Fig. 2c).

Differential accessibility analysis revealed a substantial number of differentially accessible genomic areas/peaks across all cell types, with top affected clusters being CCR2^+^ macrophages and neutrophils. Interestingly, most differential peaks were unique across cell types, suggesting that the loss of KDM6A has a unique impact in the different hematopoietic lineages (Extended Data Fig. 4f).

First, our analysis focused on CCR2^+^ recruited macrophages due to their increased infiltration into the infarcted myocardium. Aggregate ATAC−seq coverage plots revealed that, in *Kdm6a*^*Δ-Haem*^ CCR2^+^ macrophages, genomic regions diverged in accessibility compared to controls, reflecting a bidirectional shift in chromatin state (Fig. 2d). More specifically, we identified more regions with increased accessibility (625) than with decreased accessibility (263) in *Kdm6a*^*Δ-Haem*^ CCR2^+^ macrophages (Fig. 2e), suggesting a tendency toward chromatin opening. This imbalance in accessibility suggests a shift to a potentially more transcriptionally active state in the absence of KDM6A.

To assess the potential functional relevance of the observed chromatin accessibility changes, we linked differentially accessible peaks to genes based on predicted peak−gene interactions. This analysis revealed that genes involved in inflammation and myeloid cell activation (*Il1a*, *Nfkb1*, *Cd74*, *Fos*, *Junb*, *Cebpa*, *Cebpb*) were most strongly associated with regions of increased accessibility in *Kdm6a*^*Δ-Haem*^ CCR2^+^ macrophages (Fig. 2f). Gene Ontology enrichment analysis of significantly more accessible genes further supported this pro-inflammatory bias, with top enriched terms including ‘regulation of inflammatory response’, ‘cell adhesion’ and ‘response to external stimulus’ (Fig. 2g). These findings suggest that the epigenetic alterations observed in KDM6A-deficient CCR2^+^ macrophages may prime macrophages for heightened inflammatory activation after MI.

Chromatin accessibility changes additionally corresponded with altered transcription factor binding dynamics and notably increased binding of transcription factors from the AP-1 family, composed of various Jun (JUN, JUNB, JUND), Fos (FOS, FOSB, FOSL1, FOSL2) and Atf (ATF1, ATF3, ATF4, BATF) subunits as well as members of the C/EBP family (for example, CEBPA, CEBPB, CEBPE, CEBPG). Concurrently, we observed reduced binding of chromatin structural proteins such as CTCF, indicating potential disruption of chromatin architecture (Fig. 2h).

We subsequently analyzed the downstream impact of the observed epigenetic changes using scRNA-seq in CCR2^+^ recruited macrophages and Ly6c^high^ monocytes. In CCR2^+^ recruited macrophages, KDM6A deficiency led to a robust pro-inflammatory transcriptional response, with 245 genes upregulated and 34 genes downregulated compared to wild-type (Fig. 2i) as Gene Ontology term enrichment of upregulated genes revealed activation of inflammatory pathways (Fig. 2j). *Kdm6a*^*Δ-Haem*^ macrophages exhibited elevated expression of inflammatory mediators (*Il1b*, *Nlrp3*, *Cxcl2*, *Saa3*, *Fos*) alongside oxidative stress-related genes such as *Hif1a*, *Sod2* and *Txnip* (Fig. 2k), indicating a heightened inflammatory and oxidative state. Notably, regulatory genes such as *Nfkbia*, *Tnfaip3* and *Edil3* were also upregulated, suggesting the presence of compensatory mechanisms attempting to counterbalance the inflammatory activation.

**Extended Data Fig. 5:**
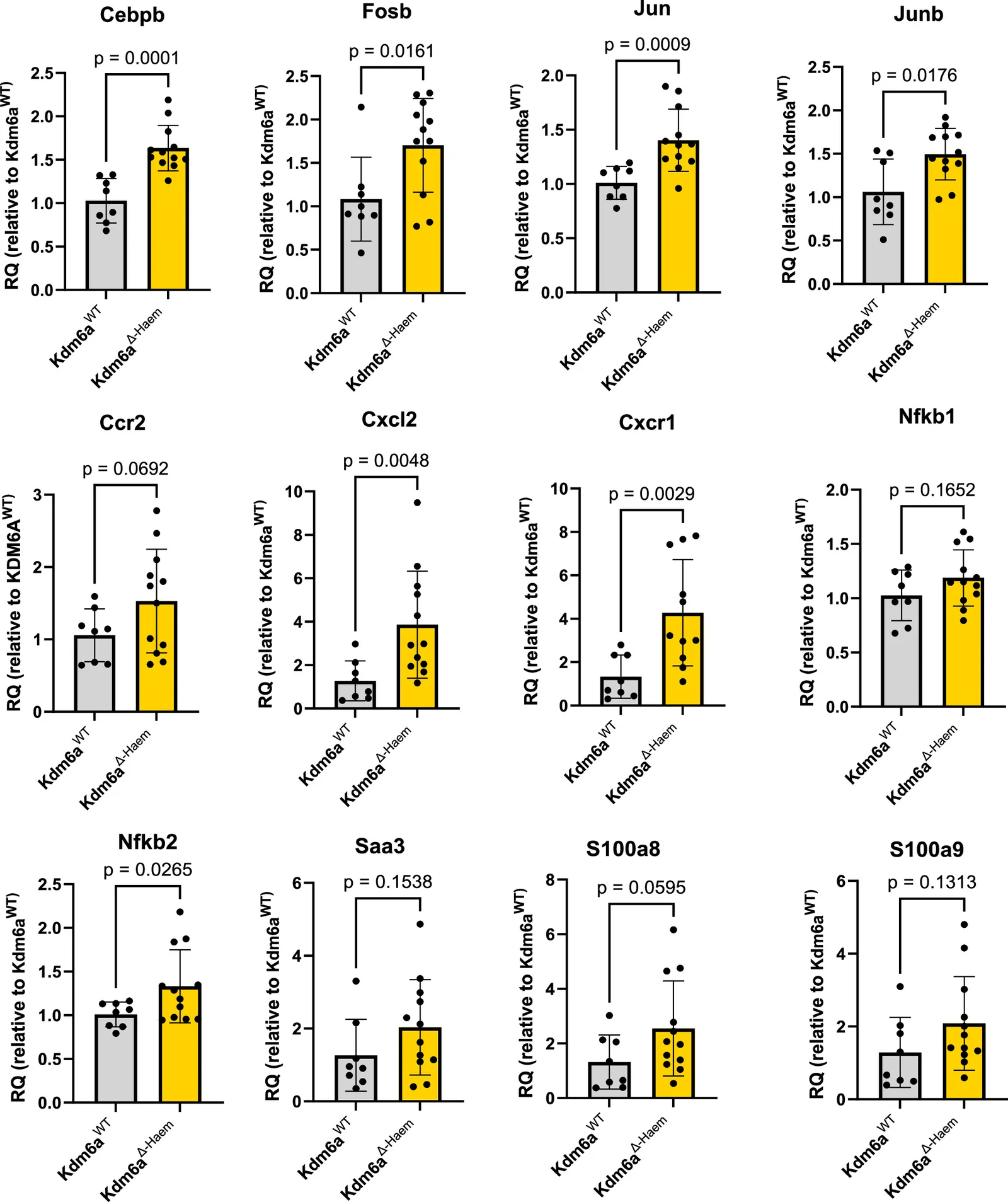
Validation of pro-inflammatory and pro-chemotactic genes identified by single-cell RNA-sequencing in the hearts of KDM6A^Δ-Haem^ mice at 3 days post-myocardial infarction. Total cardiac RNA was isolated from *KDM6A*^*WT*^ and *KDM6A*^*Δ-Haem*^ mice and processed for qPCR of genes that were identified as upregulated in single-cell RNA sequencing experiments. Statistical test two-tail Welch’s t-test. Data points represent individual biological replicates and presented as mean ± SEM. Source data

In Ly6c^high^ monocytes, precursors of differentiated macrophages, 113 genes were upregulated and 122 were downregulated in *Kdm6a*^*Δ-Haem*^ cells (Fig. 2i), indicating broader but more moderate transcriptional reprogramming. Gene Ontology analysis terms of enriched genes were dominated by glycolytic and carbohydrate catabolic processes (Fig. 2j), which were reflected in the upregulation of glycolysis-related genes (*Gapdh*, *Pkm*, *Aldoa*, *Pgk1*, *Pgam1*). This metabolic shift occurred alongside increased expression of inflammatory genes (*Il1b*, *Ccr2*, *S100a8*, *Hif1a*) and oxidative stress markers (*Sod2*, *Txnip*), suggesting that KDM6A loss promotes an inflammatory phenotype that is both metabolically and oxidatively activated. We validated the pro-inflammatory signature observed in *Kdm6a*^*Δ-Haem*^ hearts via quantitative polymerase chain reaction (qPCR) of key genes upregulated in CCR2^+^ macrophages and Ly6c^high^ monocytes, in total cardiac RNA, suggesting a generalized inflammation response (Extended Data Fig. 5).

**Extended Data Fig. 6:**
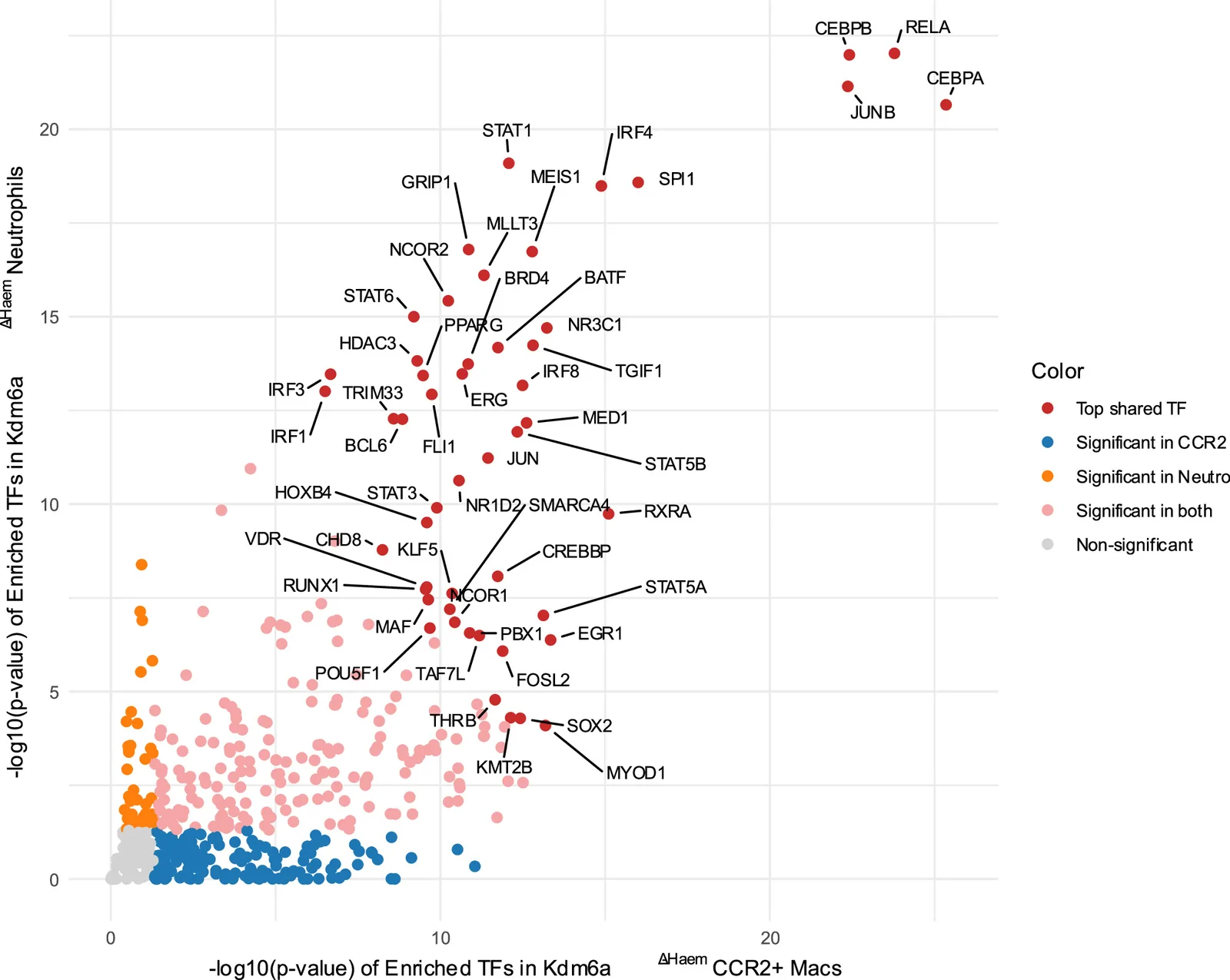
LISA analysis. Differentially upregulated genes from *Kdm6a*^*Δ-Haem*^ neutrophils and CCR2 recruited cells (CCR2 macrophages and Ly6c-high monocytes combined) were used for epigenetic Landscape In Silico deletion Analysis (LISA) to infer upstream transcription factors and/or chromatin regulators responsible for the perturbation of the input genes. Output per cell type was plotted according to significance.

To better understand the upstream regulators driving these transcriptional changes, we applied epigenetic landscape in silico deletion analysis (LISA)^9^. LISA predicted transcription factors and chromatin regulators responsible for the observed upregulated transcriptional signatures, highlighting significant enrichment for NF-κB signaling (RELA, CREBBP) and inflammatory activation (JUNB, CEBPA, CEBPB, STAT5A, STAT5B, JUN) in *Kdm6a*^*Δ-Haem*^ recruited macrophages, consistent with previous transcription factor binding analysis (Extended Data Fig. 6).

Collectively, these results demonstrate that KDM6A loss in recruited macrophages in MI induces extensive transcriptional and epigenetic reprogramming, marked by heightened inflammatory activation, disrupted chromatin architecture and a metabolic shift toward increased glycolysis.

### KDM6A deletion induces transcriptional and chromatin reprogramming toward increased inflammatory and chemotactic signatures in neutrophils after MI

**Fig. 3:**
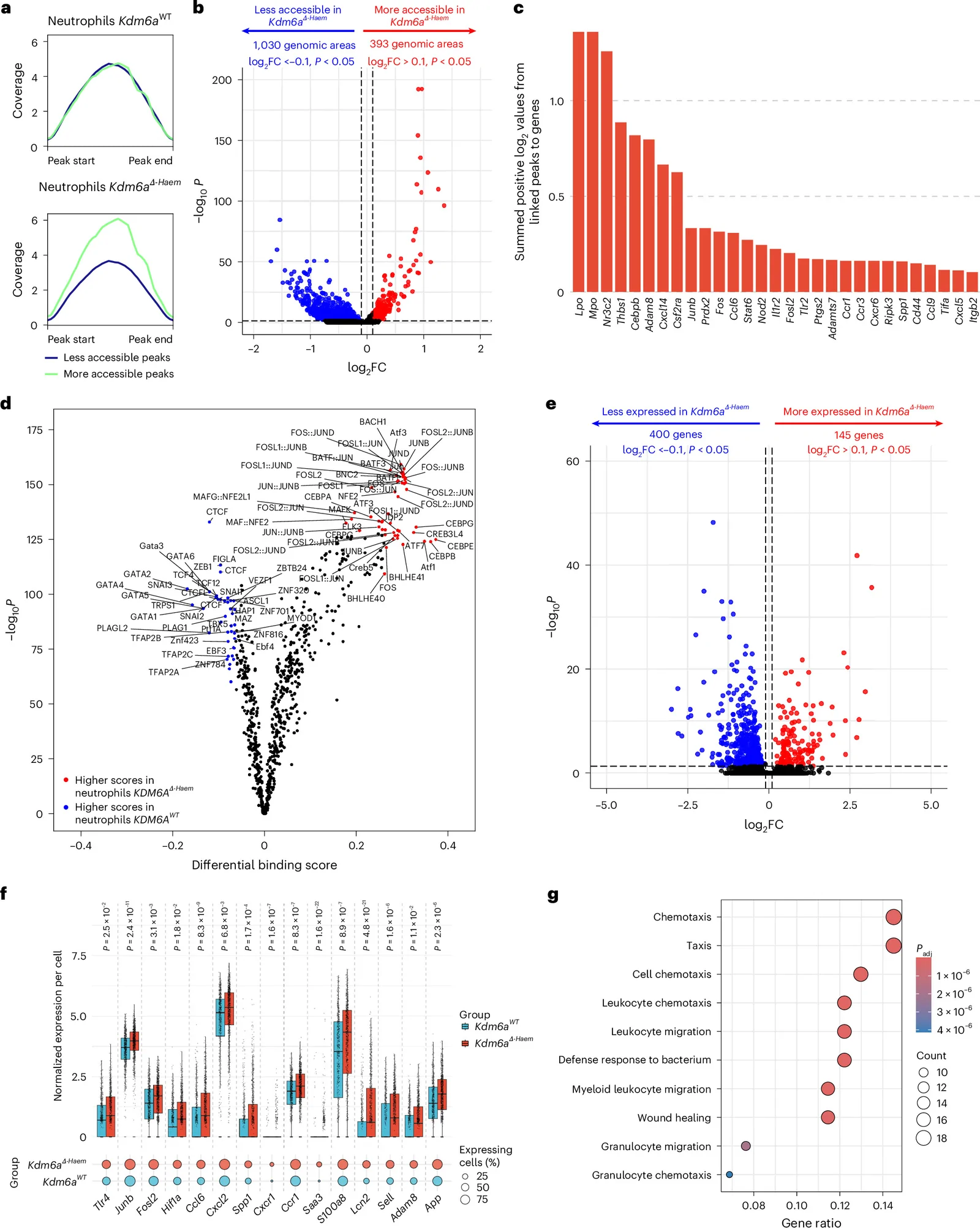
KDM6A deletion induces transcriptional and chromatin reprogramming toward increased inflammatory and chemotactic signatures in neutrophils after MI. **a**, ATAC−seq coverage plots showing average chromatin accessibility signal (average CPM) at genomic regions identified as differentially accessible peaks in neutrophils from *Kdm6a*^WT^ (top) and *Kdm6a*^*Δ-Haem*^ (bottom) mice. **b**, Volcano plot of differentially accessible peaks in *Kdm6a*^WT^ and *Kdm6a*^*Δ-Haem*^ neutrophils (peak cutoff; 10% detection in either *Kdm6a*^WT^ or *Kdm6a*^*Δ-Haem*^ group). **c**, Summed log_2_ positive fold change values from differentially accessible chromatin peaks linked to genes in *Kdm6a*^*Δ-Haem*^ neutrophils. **d**, Differential transcription factor binding activity predicted from chromatin accessibility data with the TOBIAS tool in neutrophils from *Kdm6a*^*Δ-Haem*^ compared to *Kdm6a*^WT^ mice. **e**, Volcano plots of differentially expressed genes in neutrophils (gene cutoff; 10% expression in either *Kdm6a*^WT^ or *Kdm6a*^*Δ-Haem*^ group). **f**, Normalized gene expression data from scRNA-seq per cell (top) and the percentage of expressing cells (bottom) for differentially upregulated genes in neutrophils from *Kdm6a*^*Δ-Haem*^ versus *Kdm6a*^WT^ mice. Differentially expressed genes were identified using the two-tailed Wilcoxon rank-sum test, with Bonferroni correction for multiple comparisons. Box plots show the median (center line) and the 25th and 75th percentiles (lower and upper box boundaries). Whiskers extend to the lowest and highest observations within 1.5× the interquartile range below and above the box. Data points represent individual cells derived from *n* = 3 mice per group (*Kdm6a*^WT^ versus *Kdm6a*^*Δ-Haem*^). **g**, Top enriched Gene Ontology terms enriched in differentially upregulated genes in *Kdm6a*^*Δ-Haem*^ neutrophils.

Neutrophils represented another myeloid subset significantly altered transcriptionally and epigenetically in *Kdm6a*^*Δ-Haem*^ mice after MI (Extended Data Fig. 4e,f). Neutrophils exhibited similar bidirectional chromatin remodeling to CCR2^+^ macrophages upon KDM6A loss. However, in contrast to CCR2^+^ macrophages, most differentially accessible regions became less accessible (1,030 down versus 393 up; Fig. 3a,b). Regions with increased accessibility were associated with genes related to enhanced activation of neutrophils (*Mpo*, *Lpo*, *Nr3c2*, *Thbs1*, *Cebpb*, *Nod2*, *Adam8*, *Junb*, *Fos*, *Il1r2*, *Stat6*) and chemotaxis (*Ccl6*, *Ccr1*, *Ccr3*, *Cxcr6*, *Cxcl5*, *Itgb2*, *Cd44*) (Fig. 3c).

Corresponding shifts in chromatin accessibility aligned with altered transcription factor occupancy, including enhanced binding by AP-1 and C/EBP family members (Fig. 3d), mirroring the trends observed in CCR2^+^ recruited macrophages. Interestingly, we observed diminished binding of GATA transcription factors (GATA1−5), a pattern previously linked to reduced anti-inflammatory regulation and promotion of pro-inflammatory myeloid phenotypes^10,11^.

**Extended Data Fig. 7:**
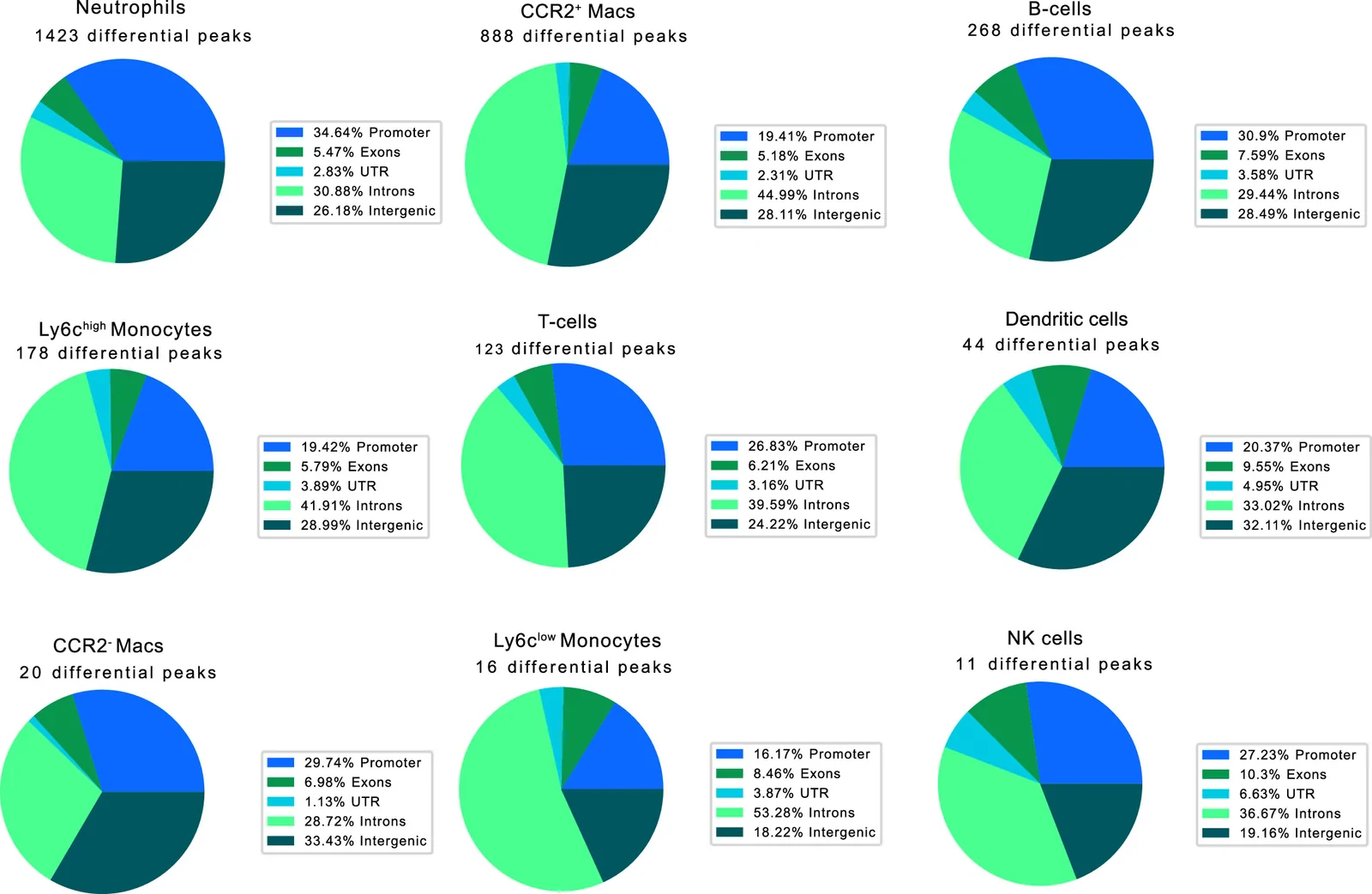
Differential peaks genomic location in *Kdm6a*^*Δ-Haem*^ mice. Genomic distribution of differentially accessible chromatin regions across immune cell subsets in *Kdm6a*^*Δ-Haem*^ mice. Each chart indicates the percentage of the differential peaks’ base pairs located within promoters, exons, untranslated regions (UTRs), introns, and intergenic regions. The number of differentially accessible peaks per cell type is indicated above each chart.

Analysis of the genomic distribution of differentially accessible regions—covering promoters, exons, introns, intergenic regions and untranslated regions—revealed widespread chromatin alterations across all cell types, indicating a broad disruption of chromatin structure but with clear cell-type-specific patterns (Extended Data Fig. 7). Notably, neutrophils exhibited prominent differential accessibility in promoters (34.64%) along with substantial changes in intronic (30.88%) and intergenic (26.18%) regions. By contrast, CCR2^+^ macrophages predominantly showed changes within intronic (44.99%) and intergenic (28.11%) regions (Extended Data Fig. 7).

To assess the transcriptional consequences of chromatin remodeling in KDM6A-deficient neutrophils, we next performed differential gene expression analysis. A total of 145 genes were significantly upregulated, whereas 400 were downregulated in *Kdm6a*^*Δ-Haem*^ neutrophils compared to control (Fig. 3e). Many of the upregulated genes were associated with chemotaxis and immune activation, including *Saa3*, *Junb*, *Ccl6*, *Cxcr1*, *Ccr1*, *S100a8*, *Sell* and *Cxcl2* (Fig. 3f). Gene Ontology term analysis of upregulated genes confirmed enrichment of pathways related to leukocyte chemotaxis and myeloid cell migration (Fig. 3g).

Collectively, these data indicate that, despite a global trend toward chromatin closure, KDM6A-deficient neutrophils selectively upregulate a focused inflammatory and migratory program, which likely contributes to their pathological role in sustained cardiac inflammation after MI.

### Dysregulated monocyte transcriptomic signatures in patients with HF with KDM6A-driven CH

**Fig. 4:**
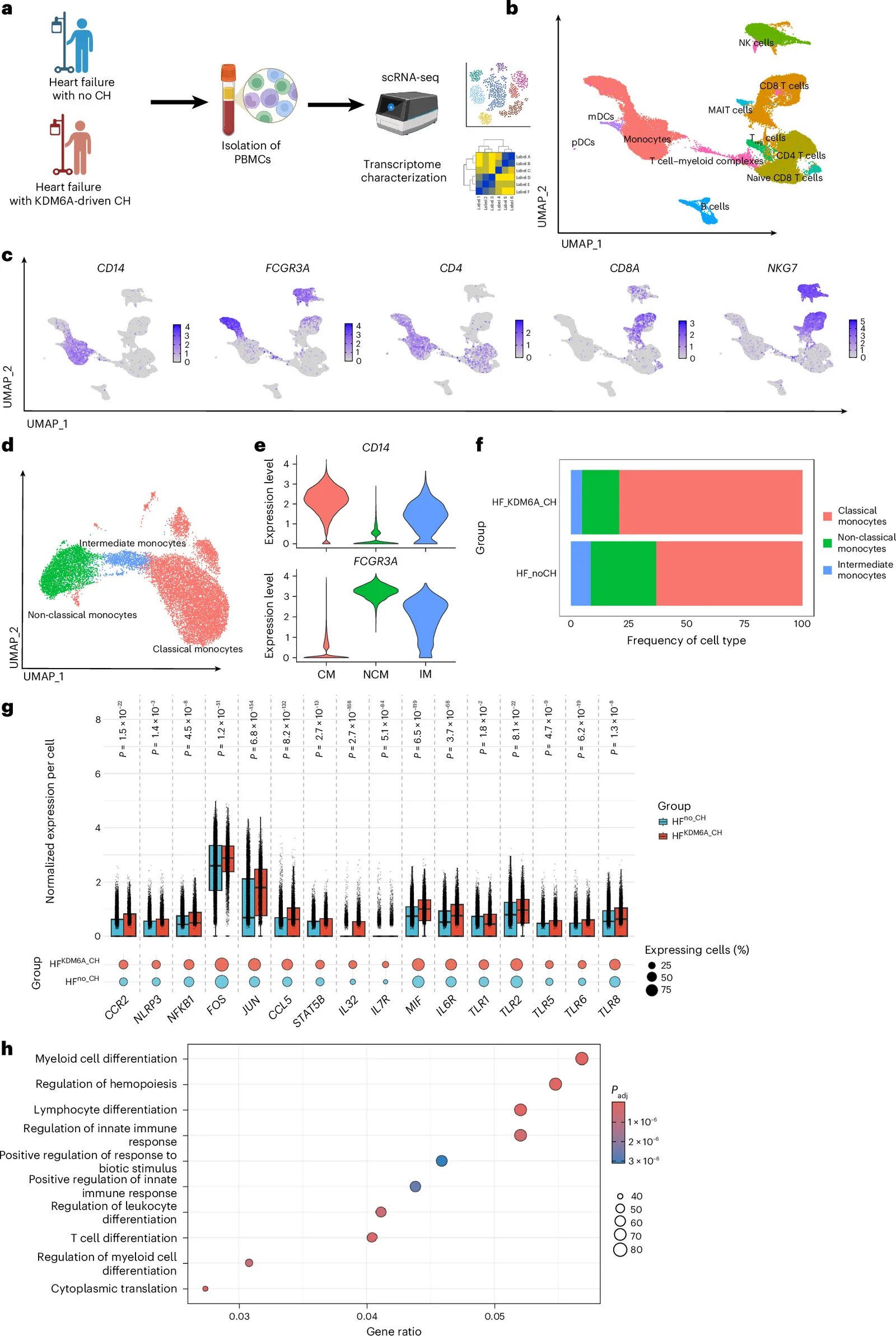
Dysregulated transcriptomic phenotype in monocytes from KDM6A CH HF. **a**, Experimental design. **b**, UMAP clustering of scRNA-seq data of PBMCs from patients with HF with and without KDM6A CH (*n* = 3 patients with HF with no CH, *n* = 2 patients with HF with KDM6A CH). **c**, UMAP plots of key peripheral leukocyte markers. **d**, UMAP of subclustered monocytes. **e**, Gene expression of *CD14* and *FCG3RA* in monocyte subclusters. **f**, Distribution of monocyte subtypes in patients with HF with and without KDM6A CH. **g**, Top enriched Gene Ontology terms enriched in differentially upregulated genes in monocytes of patients with HF with KDM6A CH. Differential gene fold cutoff for Gene Ontology term analysis was log_2_FC ≥ ±0.1, adjusted *P* < 0.05 and at least 10% gene expression in either group. **h**, Normalized gene expression data from scRNA-seq per cell (top) and the percentage of expressing cells (bottom) for differentially upregulated genes in neutrophils from patients with HF with KDM6A CH versus without CH. Differentially expressed genes were identified using the two-tailed Wilcoxon rank-sum test, with Bonferroni correction for multiple comparisons. Box plots show the median (center line) and the 25th and 75th percentiles (lower and upper box boundaries). Whiskers extend to the lowest and highest observations within 1.5× the interquartile range below and above the box. Data points represent individual cells derived from *n* = 3 patients with HF with no CH and from *n* = 2 patients with HF with KDM6A CH. Panel **a** created in BioRender; Solomonidis, E. G. https://biorender.com/ls62rrc (2026). CM, classical monocytes; IM, intermediate monocytes; NCM, non-classical monocytes; NK, natural killer; *P*_adj_, adjusted *P* value; T_reg_, regulatory T.

**Extended Data Fig. 8:**
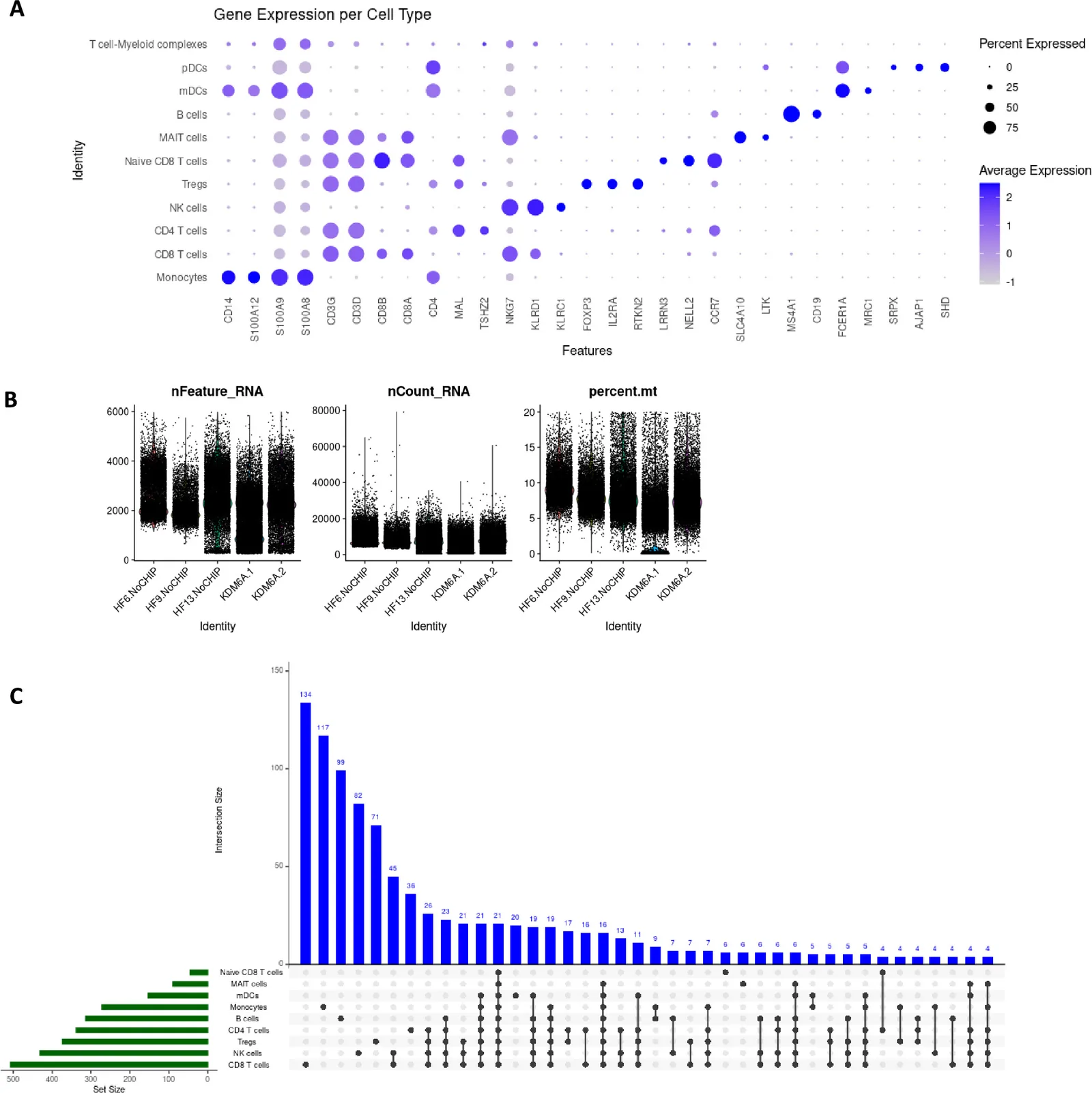
Single cell RNA-seq analysis of PBMCs from HF patients with and without KDM6A driven CH. (**A**) RNA expression of key markers used for annotation of peripheral leukocyte subsets. (**B**) Quality control metrics for single-cell RNA-seq data; genes/cells, UMIs/cells, %mitochondrial RNA/cell (**C**) Intersection plot per cell type of differentially expressed genes in HF patients with KDM6A clonal haematopoiesis (CH).

To evaluate if our mouse findings translate into clinical relevance, we profiled peripheral blood mononuclear cells (PBMCs) from patients with ischemic heart failure with reduced ejection fraction (HFrEF) with or without KDM6A-driven CH using scRNA-seq (Fig. 4a). Patient characteristics are depicted in Supplementary Table 1. Uniform manifold approximation and projection (UMAP) clustering identified major immune subsets, including monocytes, T and B cells, mucosal-associated invariant T (MAIT) cells, plasmacytoid dendritic cells (pDCs) and myeloid dendritic cells (mDCs) (Fig. 4b,c and Extended Data Fig. 8a,b).

Differential gene expression analysis revealed transcriptional differences across multiple leukocyte subsets (Extended Data Fig. 8c). Given the myeloid-specific inflammatory phenotype observed in *Kdm6a*^*Δ-Haem*^ mice, we focused our analysis on monocyte populations.

Subclustering revealed classical (CD14^+^), intermediate and non-classical (CD16^+^) monocytes (Fig. 4d,e). Notably, patients with HF with KDM6A CH showed a marked expansion of classical monocytes, the subset most associated with inflammatory responses (Fig. 4f). Differential expression analysis across all monocyte subsets identified upregulated genes in KDM6A-driven CH involved in myeloid differentiation, immune regulation and leukocyte activation (Fig. 4g). This included genes such as *CCR2*, *NFKB1*, *FOS*, *JUN*, *CCL5*, *STAT5B*, *IL32*, *IL7R* and *MIF* and multiple Toll-like receptors (*TLR1*, *TLR2*, *TLR5*, *TLR6*, *TLR8*), closely mirroring the inflammatory signatures observed in mouse *Kdm6a*^*Δ-Haem*^ myeloid cells (Fig. 4h; gene interection found in Supplementary Table 1). These results suggest that KDM6A CH in human patients with HF is associated with enhanced myeloid-driven inflammation and may contribute to disease progression.

### Altered interactions in the cardiac milieu driven by KDM6A CH monocytes

**Fig. 5:**
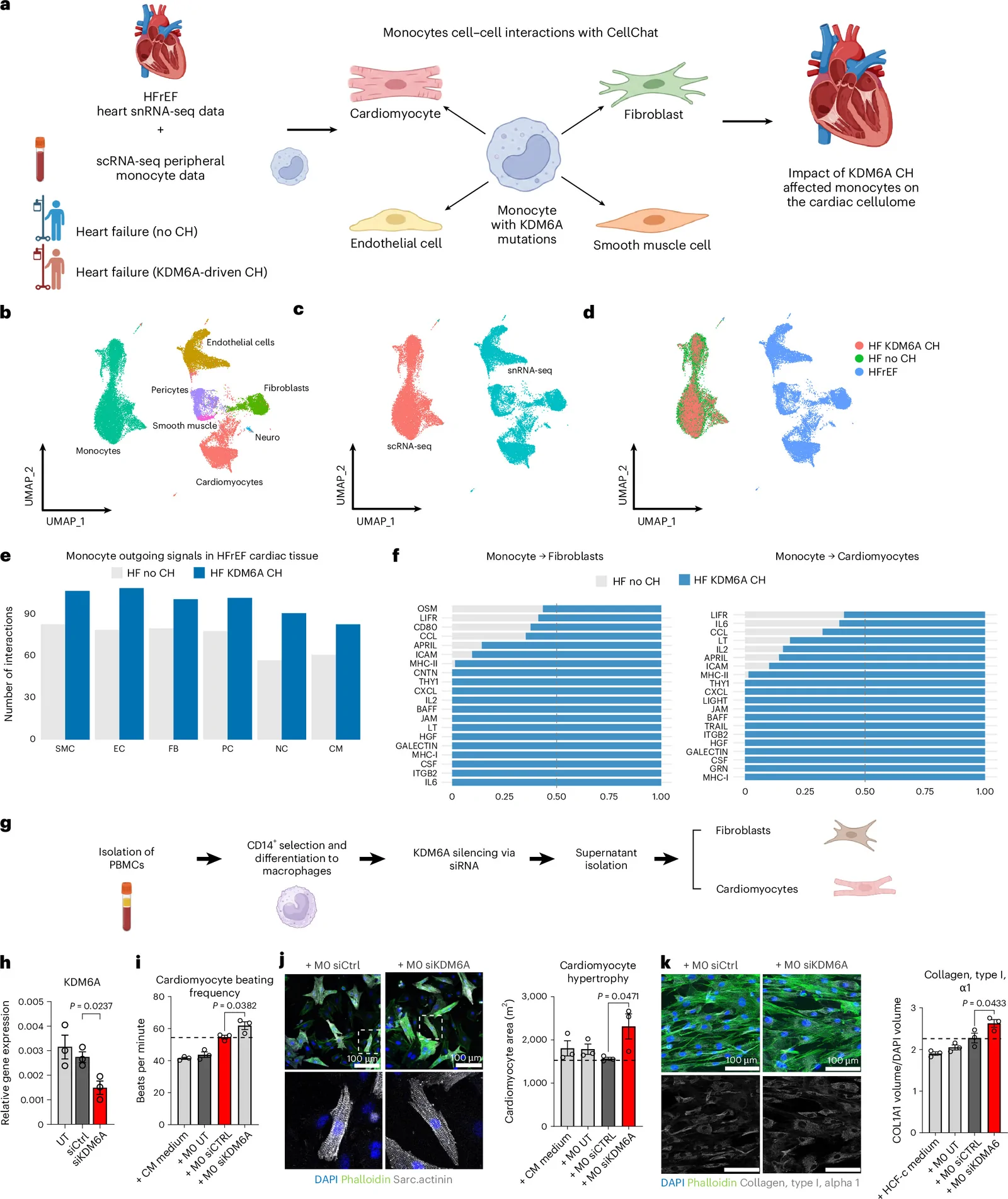
Altered interactions in the cardiac milieu driven by KDM6A CH monocytes. **a**, Analysis workflow. **b**–**d**, UMAP plots of integrated scRNA-seq of patients with HF with and without KDM6A CH and snRNA-seq of cardiac cells from *n* = 3 patients with HFrEF, here grouped by cell type (**b**), grouped by sequencing type (**c**) and grouped by group (**d**). **e**, Outgoing signals from monocyte with and without KDM6A CH toward HFrEF cardiac cells, as inferred by CellChat. **f**, Enriched signaling pathways in monocytes with KDM6A CH toward HFrEF cardiac fibroblasts and cardiomyocytes. **g**, In vitro experimental design. **h**, siRNA knockdown of KDM6A in human monocyte-derived M0 macrophages (relative gene expression, normalized to *RPLP0*, *P* = 0.024, *n* = 3 per group, unpaired two-tailed *t*-test). **i**, Rat cardiomyocyte beating frequency upon stimulation with supernatant from KDM6A-silenced M0 macrophages (*n* = 3 per group, one-way ANOVA with Dunnett’s multiple comparisons, *P* = 0.038, siCtrl versus siKDM6A). **j**, Right: representative images of rat cardiomyocytes stained with sarcomeric actinin, phalloidin and DAPI. Zoomed regions (bottom) are derived from white boxed areas. Scale bars, 100 μm. Left: quantification of cardiomyocyte area (*n* = 3 per group, one-way ANOVA with Dunnett’s multiple comparisons, *P* = 0.047, siCtrl versus siKDM6A). **k**, Human cardiac fibroblast activation assay as measured by collagen α1(I) immunostaining in fibroblasts treated with supernatant from KDM6A-silenced M0 macrophages (*n* = 3 per group, one-way ANOVA with Dunnett’s multiple comparisons, *P* = 0.043, siCtrl versus siKDM6A) and representative images (scale bars, 100 μm). Data points represent independent biological replicates. Data are represented as mean ± s.e.m. Panels created in BioRender: **a**, Solomonidis, E. G. https://biorender.com/z9v28bn (2026); **g**, Solomonidis, E. G. https://biorender.com/u4sy5ul (2026). Ctrl, control; EC, endothelial cells; FB, fibroblasts; NC, neuronal cells; PC, pericytes; SMC, smooth muscle cells; UT, untreated. Source data

**Extended Data Fig. 9:**
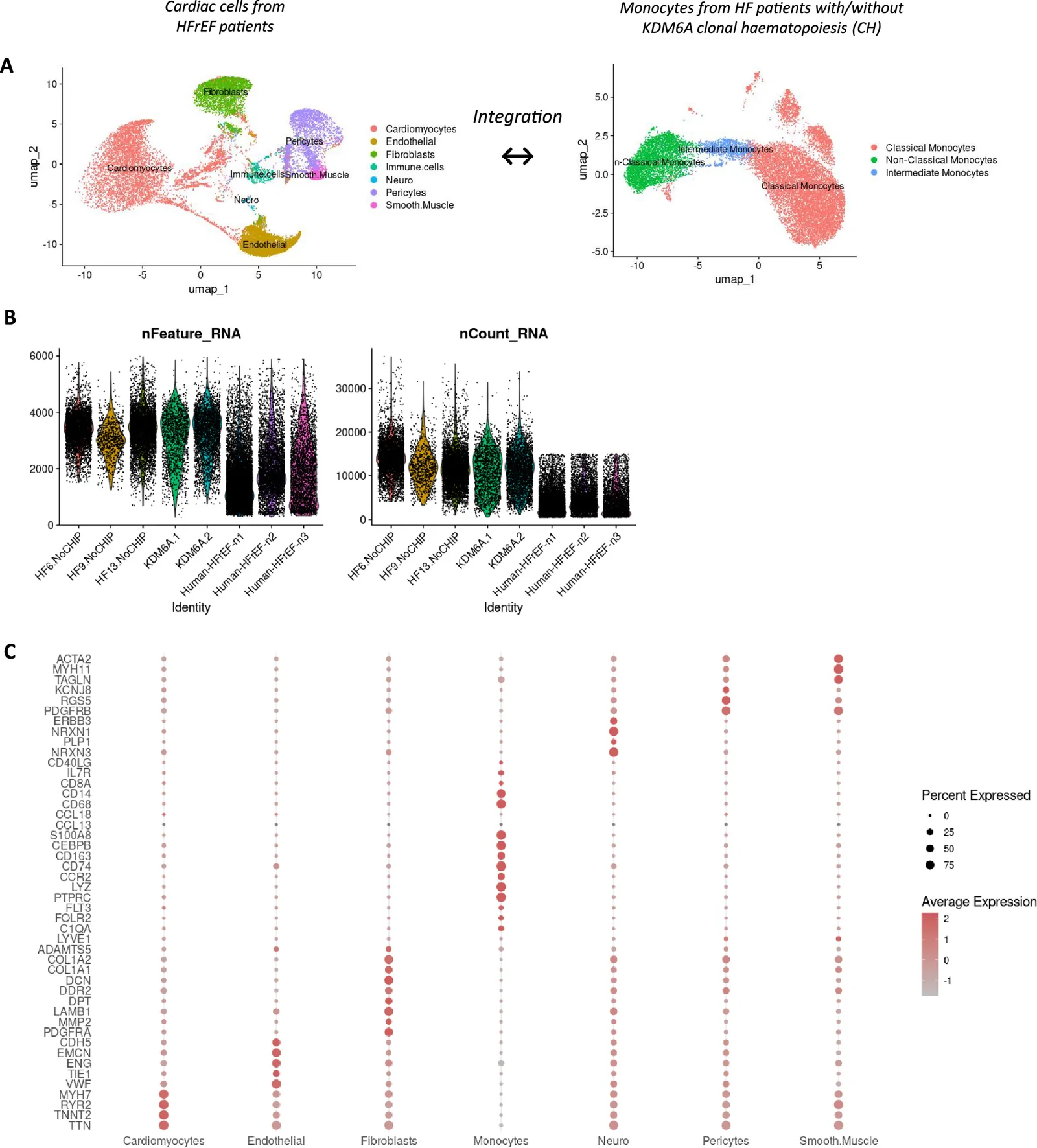
Integration of snRNAseq and scRNAseq data. (**A**) Uniform manifold approximation and projection (UMAP) clustering of single-cell RNA sequencing data from published data from heart failure patients with reduced ejection fraction (HFrEF) and UMAP clustering of subclustered monocytes of HF patients with/without KDM6A clonal haematopoiesis (CH) (**B**) Quality control metrics for filtered single-cell RNA-seq data; genes/cells, UMIs/cells, %mitochondrial RNA/cell (**C**) RNA expression of key markers used for annotation of leukocyte subsets.

To understand how KDM6A-mutated monocytes influence cardiac tissue in HF, we performed CellChat^12^ analysis by integrating monocyte scRNA-seq data from patients with HF with and without KDM6A CH and publicly available heart single-nucleus RNA sequencing (snRNA-seq) from patients with HFrEF^13^ (Fig. 5a–d and Extended Data Fig. 9a–c) (patient details published previously^14^). Cell−cell interaction modeling revealed a significant increase in inferred outgoing signaling from KDM6A CH monocytes toward multiple cardiac cell populations, including fibroblasts and cardiomyocytes (Fig. 5e). Among the top enriched pathways toward fibroblasts and cardiomyocytes were IL-6, CCL, HGF and MHC-I/II signaling, implicating both inflammatory and fibrotic communication circuits (Fig. 5f). To functionally validate these predictions, we silenced KDM6A in human monocyte-derived macrophages using small interfering RNA (siRNA) and exposed primary cardiomyocytes and cardiac fibroblasts to their conditioned media (Fig. 5g,h). Cardiomyocytes treated with KDM6A-deficient macrophage supernatant exhibited significantly increased beating frequency and hypertrophy (Fig. 5i,j). Similarly, fibroblasts exposed to the same conditioned medium showed elevated collagen α1(I) chain production, indicating enhanced fibrotic activation (Fig. 5k). These findings suggest that monocytes harboring KDM6A mutations may exacerbate HF pathology by aberrantly reshaping paracrine signaling in the cardiac microenvironment.

### Inhibition of IL-1β improves cardiac function after MI in *Kdm6a*^*Δ-Haem*^ mice

Having established that hematopoietic KDM6A loss promotes inflammatory signaling in the infarcted cardiac niche, enhances myeloid recruitment and induces macrophage-derived paracrine programs capable of activating cardiac resident cells, we next asked whether this inflammatory axis could be therapeutically intercepted. Given the prominent IL-1β-associated signature and prior links with CH-driven inflammation^15^, we tested whether IL-1β blockade could mitigate the detrimental effects of hematopoietic KDM6A loss in *Kdm6a*^*Δ-Haem*^ mice.

**Fig. 6:**
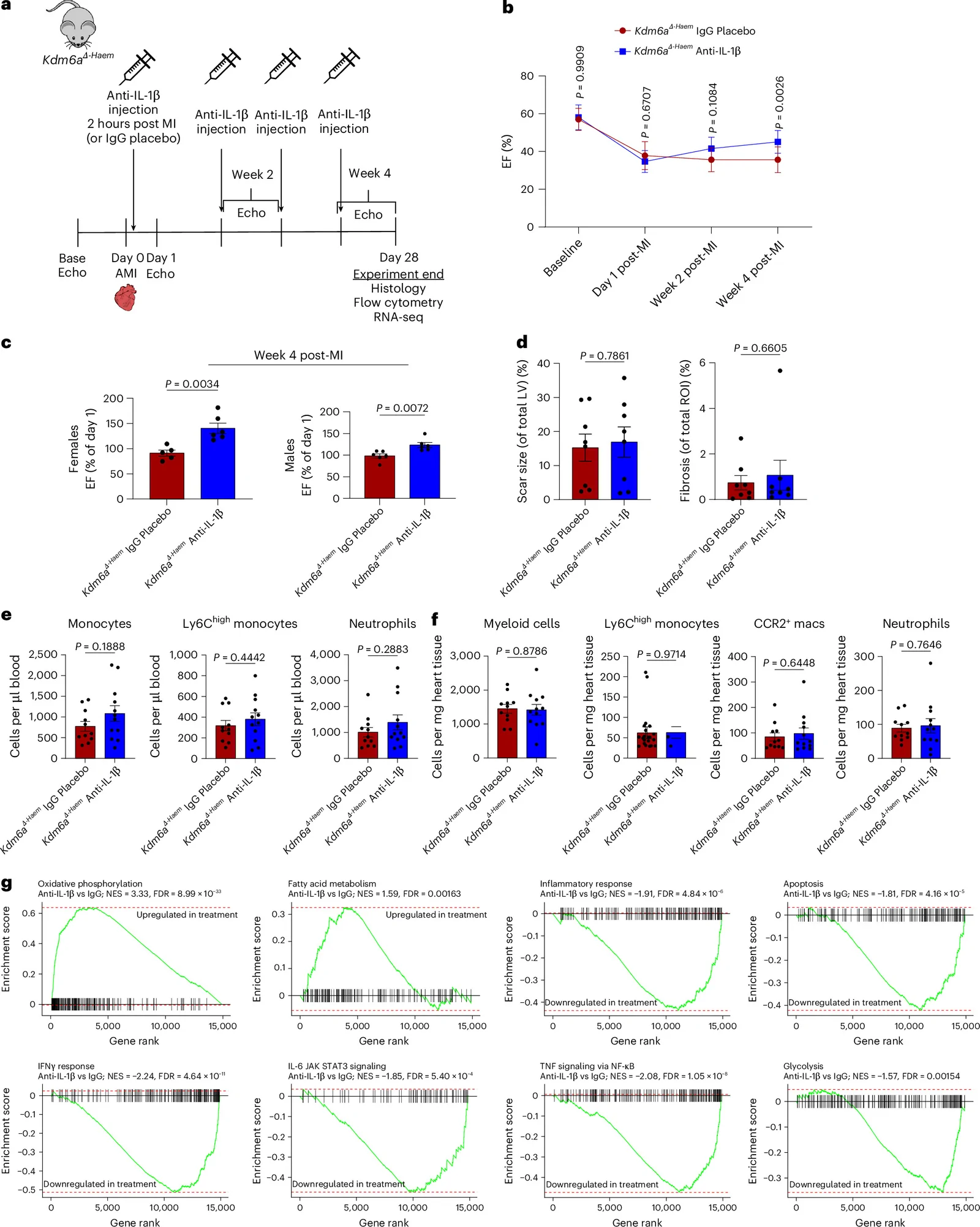
Anti-IL-1β inhibition attenuates the effects of hematopoietic KDM6A loss on cardiac recovery after MI. **a**, Experimental design. **b**, EF was measured longitudinally at baseline, day 1 after myocardial infarction (MI), 2 weeks after MI and 4 weeks after MI (*Kdm6a*^*Δ-Haem*^ IgG *n* = 11 versus *Kdm6a*^*Δ-Haem*^ anti-IL-1β *n* = 12). **c**, Four-week post-MI EF data split in treated male and female mice (male, *n* = 5 IgG versus *n* = 6 IL-1β, *P* = 0.0034; female, *n* = 6 IgG versus *n* = 6 IL-1β, *P* = 0.0072). **d**, Left ventricular scar size area and interstitial fibrosis analysis (*n* = 8 IgG versus *n* = 8 IL-1β). **e**, Peripheral blood myeloid cell analysis with flow cytometry at 28 days after MI in treated mice (IgG *n* = 11 versus anti-IL-1β *n* = 12). **f**, Myeloid cell analysis in the myocardium using flow cytometry at 28 days after MI in treated mice (IgG *n* = 11 versus anti-IL-1β *n* = 12). **g**, Gene set enrichment analysis in RNA-seq data from anti-IL-1β-treated hearts at day 28 after MI (*n* = 6 IgG versus *n* = 6 IL-1β). Timecourse EF data were analyzed using two-way repeated-measures ANOVA with treatment and time as factors, followed by Sidak’s multiple-comparisons test to compare groups within each timepoint. Statistical analysis in sex-split EF analysis, histology and flow cytometry was done with a two-tailed unpaired *t*-test. Data points in graphs represent individual biological replicates and are presented as mean ± s.e.m. NES, normalized enrichment score. Credit: syringe and mouse icons in **a** adapted from Openclipart under a Creative Commons licence CC0 1.0. Source data

*Kdm6a*^*Δ-Haem*^ mice were treated with a neutralizing anti-mouse IL-1β antibody at 2 hours after MI, followed by weekly intraperitoneal injections for a total of four injections until euthanization at 4 weeks after MI. Control animals received isotype immunoglobulin G (IgG) using the same treatment schedule. Echocardiographic assessment was performed at day 1, week 2 and week 4 after MI to determine whether IL-1β inhibition could improve cardiac functional recovery after injury in *Kdm6a*^*Δ-Haem*^ mice (Fig. 6a).

**Extended Data Fig. 10:**
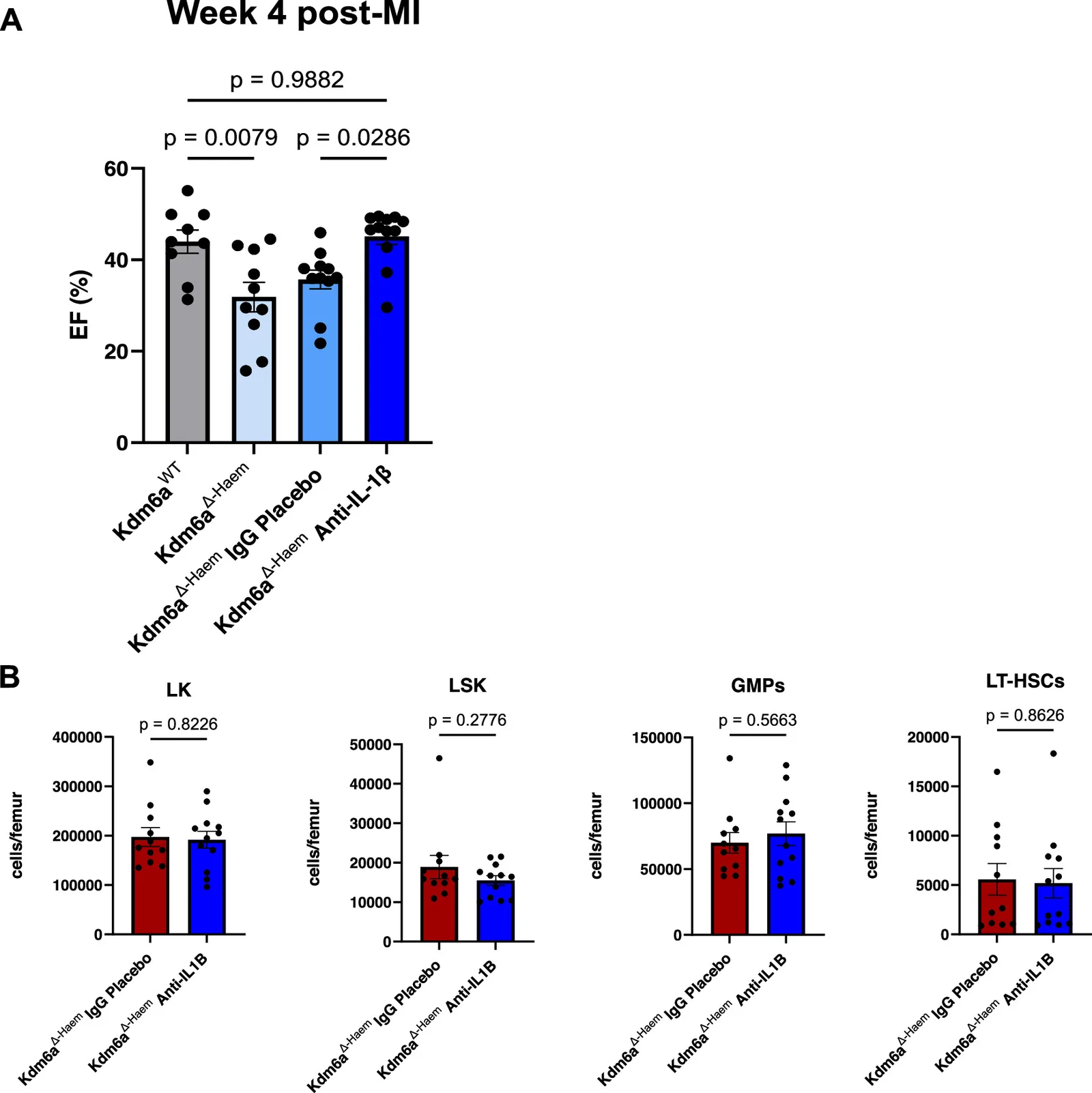
Anti-IL-1β inhibition rescues the effects of haematopoietic KDM6A loss on cardiac function post-MI. **(A)** Original ejection fraction (EF) data at 4 weeks post-myocardial infarction of KDM6A^WT^ vs KDM6A^Δ-Haem^ plotted alongside KDM6A^Δ-Haem^ IgG placebo and KDM6A^Δ-Haem^ anti IL-1β treatment groups. One-way ANOVA statistical test with Tukey’s multiple comparison tests. (**B**) Haematopoietic progenitor composition in the bone marrow of Kdm6a^Δ-haem^ mice at 28 days post-MI with flow cytometry. Statistical analysis was done with a two-tail unpaired t-test. Data points in all graphs represent individual biological replicates and presented as mean ± SEM. Source data

At day 1 after MI, anti-IL-1β-treated and IgG-treated *Kdm6a*^*Δ-Haem*^ mice showed a similar reduction in EF, indicating a similar initial injury burden between groups. By contrast, at week 2, there was a divergence in the cardiac function between the two groups, and, at week 4 after MI, anti-IL-1β-treated mice showed a significant improvement in EF compared to IgG-treated controls (Fig. 6b). This therapeutic benefit was observed in both male and female *Kdm6a*^*Δ-Haem*^ mice (Fig. 6c). Notably, when compared to our original post-MI dataset, IL-1β blockade resulted in a near-complete rescue of the cardiac functional phenotype caused by hematopoietic KDM6A loss, with EF approaching the levels observed in *Kdm6a*^WT^ animals 4 weeks after MI (Extended Data Fig. 10a).

Histological analysis at the 4-week terminal timepoint did not reveal significant differences in scar size or interstitial fibrosis between anti-IL-1β-treated and IgG-treated mice (Fig. 6d). We interpret this to indicate that the improvement in EF was not primarily driven by reduced infarct scar formation but, rather, by improved function of the surviving myocardium and/or attenuation of pathological inflammatory signaling during post-MI remodeling. Day 28 flow cytometric analysis did not show persistent differences in circulating monocytes, cardiac Ly6C^+^ monocytes or cardiac CCR2^+^ macrophages between anti-IL-1β-treated and IgG-treated mice (Fig. 6e,f). This likely reflects the late timepoint analyzed, because the major inflammatory leukocyte recruitment wave after MI occurs earlier. Day 28 bone marrow progenitor profiling did not show differences in LK, LSK, GMP and LT-HSC populations between anti-IL-1β-treated and IgG-treated mice (Extended Data Fig. 10b).

As female *Kdm6a*^*Δ-Haem*^ mice displayed the most pronounced impairment in cardiac function after MI in our original echocardiographic analysis, we selected female mice for bulk cardiac RNA-seq to assess the molecular effects of IL-1β blockade on the KDM6A-loss phenotype. Bulk cardiac RNA-seq from female *Kdm6a*^*Δ-Haem*^ mice at 4 weeks after MI revealed downregulation of key inflammatory pathways after anti-IL-1β treatment, such as TNF, IL-6 and IFNγ, as well as apoptosis. In parallel, pathways related to oxidative phosphorylation and fatty acid metabolism were increased, whereas glycolytic signatures were reduced (Fig. 6g). These data suggest that IL-1β blockade attenuates the sustained inflammatory stress induced by hematopoietic KDM6A loss and is associated with partial restoration of myocardial energy metabolism programs. This is relevant because adverse post-MI and HF remodeling are characterized by mitochondrial dysfunction and impaired substrate oxidation, which reduce the capacity of the myocardium to convert chemical energy into mechanical work^16,17^. Therefore, the reduction in inflammatory pathways and glycolysis, together with increased oxidative phosphorylation and fatty acid metabolism signatures, provides a molecular correlate of the improved EF observed after anti-IL-1β treatment.

Together, these data provide direct functional evidence that the detrimental effects of hematopoietic KDM6A loss after MI can be mitigated by anti-inflammatory therapy. More specifically, they identify IL-1β-dependent inflammation as a therapeutically actionable downstream component of the KDM6A-loss phenotype.

## Discussion

Here we demonstrate that lack of hematopoietic KDM6A is detrimental in the context of HF. We show that loss of KDM6A function in hematopoietic cells results in worsened cardiac function after MI through an enhanced inflammatory activation of myeloid cells as shown by increased production of pro-inflammatory cytokines. Hematopoietic KDM6A deletion results in a chromatin landscape rewiring that promotes binding of transcription factors such as AP-1 that drive expression of pro-inflammatory programs. These findings are corroborated by human HF patient data, where patients with HF with mutated KDM6A-driven CH show enhanced inflammatory signaling in circulatory monocytes with a potential to aberrantly activate cardiac cells such as fibroblasts and cardiomyocytes upon infiltration, reinforcing the link between KDM6A mutations and worsened cardiovascular outcomes. Notably, IL-1β blockade largely rescued the adverse cardiac functional phenotype in *Kdm6a*^*Δ-Haem*^ mice, providing direct evidence that the inflammatory program induced by hematopoietic KDM6A loss is not only associated with impaired cardiac recovery but also represents a therapeutically targetable mechanism.

Additionally, hematopoietic deficiency of KDM6A results in worse EF after MI, which is associated with increased recruitment of myeloid cells to the cardiac tissue. These recruited myeloid cells exhibit a pronounced pro-inflammatory phenotype, as evidenced by the upregulation of canonical inflammatory and pro-recruitment markers, including IL-1B, NLRP3 and CCR2, all of which are associated with adverse cardiovascular outcomes^18–20^. In addition to local cardiac inflammation, *Kdm6a*^*Δ-Haem*^ mice showed evidence of an amplified systemic inflammatory/remodeling response after MI, which supports the concept that hematopoietic KDM6A loss not only alters the inflammatory state within the injured myocardium but also amplifies circulating inflammatory and tissue remodeling signals during the acute post-MI response. Interestingly, we show that *Kdm6a*^*Δ-Haem*^ mice displayed an altered bone marrow progenitor compartment before injury, including increased GMPs, whereas mature myeloid cell abundance in blood and uninjured heart remained unchanged, indicating that hematopoietic KDM6A loss establishes an imbalanced or primed progenitor state, although these GMP numbers were no longer increased at the day 3 inflammatory peak. This suggests that the acute post-MI myeloid phenotype is unlikely to be explained solely by numerical expansion of the GMP pool but may, instead, reflect altered progenitor dynamics, mobilization, differentiation kinetics or responsiveness to injury-induced inflammatory cues.

Furthermore, KDM6A-deficient immune cells display dysregulated communication toward other cardiac cell types, which may further drive attenuated cardiac healing after MI. In-depth analysis of the inflammatory profile of KDM6A-deficient myeloid cells reveals concurrent upregulation of transcription factors from the NF-κB and AP-1 families, including Jun and Fos transcription factors, which act upstream of classical pro-inflammatory cytokines^21^ and are linked to aggravated cardiac inflammatory response and poor cardiac remodeling after MI^22^. In parallel, these cells display a metabolic shift toward enhanced glycolysis, a hallmark feature of myeloid cell inflammatory activation^23^. Interestingly, despite the heightened pro-inflammatory response, KDM6A-deficient myeloid cells also exhibit increased expression of anti-inflammatory genes, including NF-κB regulatory elements and *EDIL3* (also known as *DEL1*), which encodes a molecule implicated in the resolution of inflammation^24^. This suggests an intrinsic, yet dysregulated, attempt by these cells to counterbalance their exacerbated immune activation.

Moreover, chromatin accessibility profiling of KDM6A-deficient myeloid cells reveals profound alterations in chromatin structure. A substantial number of genomic regions exhibit increased accessibility, whereas others were repressed, collectively resulting in an overall chromatin landscape rewiring that favors the binding of pro-inflammatory transcription factors, such as members of the AP-1 and C/EPB families. Given that KDM6A is classically recognized as a transcriptional activator, these findings appear counterintuitive. However, they underscore the complex regulatory role of KDM6A in immune cell chromatin remodeling after MI, suggesting potential secondary effects mediated by other chromatin-modifying factors in its absence. Notably, KDM6A-deficient CCR2^+^ recruited macrophages exhibit a greater proportion of chromatin opening compared to neutrophils, which show more repressed genomics areas. Additionally, although most differentially accessible regions are unique to each cell type, indicating that KDM6A loss remodels the chromatin landscape in a lineage-specific manner, both KDM6A-deficient CCR2^+^ macrophages and neutrophils act via similar transcription factor programs, particularly AP-1 and C/EBP. This convergence in upstream regulatory networks is accompanied by a shared biological outcome of heightened expression of inflammatory and chemotactic genes.

Histone demethylases are important in immunity^25^ and implicated in the regulation of CVD^26^. KDM6A is associated with inflammation, albeit not in the context of CVD. KDM6A deficiency in the urothelium led to activated cytokine and chemokine pathways in the context of TP53-induced bladder cancer^27^. However, KDM6A contributes to the innate response during inflammation by promoting IL-6 and IFNβ production in lipopolysaccharide (LPS)-treated mouse peritoneal macrophages^28^. These contrasting observations suggest that the role of KDM6A during inflammation may differ depending on context, severity or the specific cell type involved and may further diverge when comparing partial loss versus complete ablation of KDM6A function. Recently, KDM6A was found to regulate immune responses in the context of multiple myeloma by binding and regulating genes associated with immune recognition and cytokine signaling, such as NLRC5 and CIITA^29^. Moreover, KDM6A and KDM7A bind the NF-κB binding site in TNF-induced endothelial cells, which suggests that removal of repressive histone marks by KDM6A is critical for NF-κB-dependent regulation of genes that control inflammation^30^. When using an inhibitor against KDM6A (GSK-J1), it has been shown that there is a reduction in LPS-induced pro-inflammatory cytokine production by human primary macrophages^31^. However, there is conflicting evidence regarding the regulatory role of KDM6A in inflammation as certain studies have shown that, upon inhibition of KDM6A, there is improvement in diabetes-induced renal abnormalities in an animal model of type 2 diabetes by reducing inflammation^32^. In summary, KDM6A plays a major role in immune responses and the regulation of inflammation, but the effects of KDM6A on inflammation are complex and context dependent.

Furthermore, our human data provide important clarity by directly linking KDM6A-driven CH to pathogenic inflammation in HF. Monocytes from patients harboring KDM6A mutations exhibit upregulation of key inflammatory mediators, including *NLRP3*, *NFKB1*, *FOS* and *JUN*, closely mirroring the transcriptional profiles seen in *Kdm6a*^*Δ-Haem*^ mice. These shared signatures point to a conserved mechanism of immune activation and support a role for KDM6A in restraining myeloid-driven inflammation in the context of HF. Our findings suggest potential implications for personalized medicine, particularly regarding anti-cytokine therapies for patients with KDM6A-driven CH. This is supported by recent data from a follow-up analysis of the CANTOS^33^ randomized clinical trial, which suggested that patients with MI harboring TET2-driven CH could respond better to canakinumab, targeting IL-1β, compared to those without CH^15^. In fact, our anti-IL-1β blocking experiments after MI now provide functional evidence that this inflammatory axis is indeed therapeutically targetable also in KDM6A-driven CH, as IL-1β blockade rescued the adverse cardiac phenotype caused by hematopoietic KDM6A loss after MI. More specifically, we show that *Kdm6a*^*Δ-Haem*^ mice that receive anti-IL-1β therapy after MI show a downregulation of inflammatory pathways after anti-IL-1β treatment, such as TNF, IL-6 and IFN, as well as reduction in glycolytic signatures, whereas oxidative phosphorylation and fatty acid metabolism were increased. Together, this suggests that IL-1β blockade improves cardiac recovery in *Kdm6a*^*Δ-Haem*^ mice by suppressing maladaptive inflammatory signaling caused by hematopoietic KDM6A deficiency and improving the molecular state of the surviving myocardium. This interpretation is supported by prior mouse studies in which IL-1β blockade improved left ventricular systolic function and remodeling after MI^34^, reduced post-MI emergency hematopoiesis and leukocyte accumulation^35^ and reduced myocardial inflammation without necessarily changing infarct size^36^.

Clinically, these findings are consistent with the broader concept that CH may identify inflammatory cardiovascular subgroups that could benefit from personalized anti-inflammatory therapy. This concept is now being tested prospectively in the TECTONIC trial (NCT06691217, ongoing clinical trial^37^), which evaluates whether IL-1β inhibition with canakinumab reduces vascular inflammation in patients with coronary artery disease with and without TET2-driven CH. More broadly, recent analyses of colchicine suggest that systemic anti-inflammatory therapy may also influence CH clone dynamics, with low-dose colchicine associated with attenuated clonal expansion, particularly in TET2-mutant CH^38^. Together, these studies support the emerging idea that CH status and CH genotype can become useful biomarkers for cardiovascular personalized medicine and for selecting anti-inflammatory strategies. Although no KDM6A-specific anti-inflammatory clinical trial currently exists, our data suggest that KDM6A-driven CH represents another genetically defined inflammatory CH subtype and that targeting IL-1β-dependent signaling after MI with drugs such as canakinumab^33^ or anakinra^39^ may be therapeutically relevant in the context of CH.

Certain limitations should be considered when interpreting this study. First, the relatively small patient cohort for scRNA-seq analysis may restrict the generalizability of our findings; larger, diverse cohorts would strengthen our conclusions. Moreover, although the *Kdm6a*^*Δ-Haem*^ mouse model provided valuable mechanistic insight into KDM6A-driven inflammation in HF, it is important to acknowledge the clinical heterogeneity of KDM6A mutations. Previously reported mutations to date appear to result in loss of function or partial loss of function, supporting the relevance of our knockout model^40–47^. However, because several mutations are missense variants, their precise functional consequence cannot be assigned without experimental validation. Notably, KDM6A variants reported in CH are distributed across the protein, including regions implicated in condensate formation and the C-terminal JmjC demethylase domain, rather than clustering exclusively within one functional domain. Therefore, individual KDM6A mutations may affect distinct molecular functions of the protein. This is particularly relevant because KDM6A acts through both demethylase-dependent and demethylase-independent mechanisms. KDM6A was originally characterized as an H3K27me2/3 demethylase, but it also regulates enhancer function through interactions with MLL3/MLL4-COMPASS and p300, influencing H3K4me1 and H3K27ac deposition, and has been shown to form chromatin-associated condensates that contribute to its tumor-suppressive activity^48^. Thus, complete KDM6A loss is expected to disrupt multiple layers of chromatin regulation, including H3K27 demethylation and enhancer regulation. By contrast, patient-specific missense or in-frame deletion variants may selectively impair some of these activities while sparing others. Consistent with this complexity, KDM6A loss has been shown to alter enhancer architecture, including aberrant activation of super-enhancers, supporting the idea that KDM6A deficiency can reshape transcriptional networks in ways that are not explained solely by loss of catalytic demethylase activity^47^.

The X-linked biology of KDM6A adds an additional layer of complexity. KDM6A escapes X chromosome inactivation while males express the Y-linked homolog UTY. UTY has markedly reduced enzymatic activity compared to KDM6A but may retain demethylase-independent functions. Our observation that female *Kdm6a*^*Δ-Haem*^ mice displayed a more pronounced cardiac phenotype after MI, whereas male mice were still affected, is consistent with partial compensation by UTY in males. Whether this compensation involves residual chromatin-regulatory, scaffolding or condensate-associated functions remains unresolved. Conversely, studies of hematopoietic loss of chromosome Y indicate that loss of Y-linked genes can itself influence cardiac remodeling and inflammatory disease phenotypes, suggesting that UTY and other Y-linked factors may have independent roles in cardiovascular pathology^49,50^.

Currently, it remains unresolved why KDM6A loss promotes pro-inflammatory programs after MI. One possibility is that KDM6A loss establishes epigenetic priming during hematopoietic differentiation, creating progenitor-derived myeloid cells that are more responsive to inflammatory cues within the injured myocardial niche. Hematopoiesis is a multistage differentiation process, and KDM6A-dependent chromatin alterations in progenitors may, therefore, propagate into downstream monocytes, macrophages and neutrophils, where they become functionally evident during myocardial injury. These responses could involve genome-wide chromatin dysregulation affecting enhancer−promoter interactions, possibly mediated by changes in H3K27me3, H3K4me1, H3K27ac, super-enhancer activity or condensate-dependent chromatin organization. Future studies addressing epigenetic priming in hematopoietic progenitors, functional consequences of patient-specific KDM6A mutations and targeted anti-inflammatory interventions will be important to clarify these mechanisms and to further develop personalized therapeutic strategies for patients with KDM6A-driven CH.

In summary, our findings reveal a pivotal role for KDM6A in orchestrating immune cell responses during HF after MI. Hematopoietic KDM6A loss primes inflammatory myeloid programs, amplifies systemic and cardiac inflammation, disrupts immune−cardiac crosstalk and impairs cardiac recovery after myocardial injury. The rescue of cardiac function by IL-1β blockade highlights the therapeutic potential of modulating inflammatory pathways in KDM6A-driven CH and, more broadly, in pro-inflammatory CH-associated CVD.

## Methods

The study adheres to all relevant ethical guidelines, was approved by the local ethics review board (Ethikkommission des Fachbereichs Medizin der Goethe-Universität) and complies with the Declaration of Helsinki. Consent for patient sample processing and data collection and analysis was collected as part of the protocol approved by the ethics committee of University Hospital Frankfurt. All animal experiments were conducted according to the principles of laboratory animal care and according to German national laws. Animal studies were approved by the local ethics committee (Regierungspräsidium Darmstadt, Hessen) in applications FU/1239 and FU/2124.

### Animals

*VaviCre* (*B6.Cg-Commd10*^*Tg(Vav1-icre)A2Kio*^/J; The Jackson Laboratory) heterozygous male mice were bred with female *Kdm6a*^fl/fl^ (*B6;129S-Kdm6a*^*tm1.1Kaig*^/J; The Jackson Laboratory) homozygous mice to establish *VaviCreKdm6a*^fl/fl^ offspring with a hematopoietic-specific KDM6A knockout (referred to in this paper as *Kdm6a*^*Δ-Haem*^ mice). Control mice (referred to in this paper as *Kdm6a*^WT^ mice) were littermates lacking the *VaviCre* transgene. Phenotyping experiments included both male and female mice. Single-cell RNA/ATAC experiments included female mice only. Mice were kept in a 12-hour day cycle and housed in individual ventilated cages. Mice were held at 23 °C ambient temperature and 60% humidity.

### Genotyping

Genotyping was conducted with DNA isolated from ear punches using touchdown PCR according to the protocols provided by The Jackson Laboratory for the respective mouse strains. In brief, the presence of the *VaviCre* transgene was validated, followed by validation of the presence of the *Kdm6a*^fl/fl^ transgene. Primer sequences can be found in Supplementary Table 1.

### MI model (LAD coronary artery ligation)

The animals were sedated using isoflurane, and pain relief was provided through an intraperitoneal injection of buprenorphine (0.1 mg kg^−1^ body weight) along with an intercostal nerve block using bupivacaine (1 mg kg^−1^ body weight, 0.25% concentration). For postoperative pain management, buprenorphine and carprofen (5 mg kg^−1^ body weight) were administered every 12 hours or 24 hours for 3 days. To prevent infection after surgery, ampicillin (100 mg kg^−1^ body weight) was added to the drinking water. The MI was induced by permanently ligating the LAD coronary artery while the animals were under mechanical ventilation.

### Inhibition of mouse IL-1β after MI

Mouse IL-1β was inhibited after MI via intraperitoneal injection of InVivoMAb anti-mouse/rat IL-1β antibody (Armenian hamster IgG isotype, clone B122; Bio X Cell, BE0246) at a dosage of 10 mg kg^−1^. Control animals were given InVivoMAb polyclonal Armenian hamster IgG (Bio X Cell, BE0091) at the same dosage. Injection volume was 100 μl, and the dilutant was InVivoPure pH 7.0 dilution buffer (Bio X Cell). Mice were given a total of four injections after MI—the first one 2 hours after MI and then three more on a weekly basis before termination of the experiment at 4 weeks after MI.

### Echocardiography

Echocardiographic assessments were performed using the Vevo 3100 system (Fujifilm) under isoflurane anesthesia at baseline and day 28 after MI. The acquired images were analyzed in a blinded manner with VevoLab 5.5.0 software. Both systolic and diastolic phases were evaluated by capturing images from the short and long axes of the ventricles to determine EF and left ventricular mass. The left atrial size was measured in the four-chamber view during the end-systolic phase with the mitral valve in the closed position. Diastolic function was assessed by examining the velocity of blood flow through the mitral valve using pulsed Doppler, and the mitral valve annulus motion was evaluated with tissue Doppler imaging. From these measurements, the E/A and E/e′ ratios were calculated.

### Mouse tissue processing

#### Peripheral blood

Mouse blood was isolated from euthanized mice from the vena cava after culling via cervical dislocation under isoflurane anesthesia or sampled from live mice via the tail vein prior to MI. Red blood cells were lysed with 1× RBC lysis buffer (BioLegend), after which point samples were processed for flow cytometric analysis (‘Flow cytometry analysis of mouse tissue’).

#### Heart

Mice were euthanized using cervical dislocation under isofluorane anesthesia. Hearts were perfused via the left ventricle with cold PBS and excised before being dissected and enzymatically digested (in RPMI 1640 media; 450 U ml^−1^ Collagenase I, 125 U ml^−1^ Collagenase XI, 60 U ml^−1^ DNase I and 60 U ml^−1^ hyaluronidase, 30 minutes, 37 °C). After digestion, mechanical dissociation was carried out (gentleMACS), and cells were filtered through a 40-μm nylon mesh to obtain a single-cell suspension.

#### Bone marrow

Femurs were isolated from euthanized mice. After removal of the epiphyses, the bone marrow was flushed with cold buffer of 5% FBS, 1 mM EDTA (in DPBS). The cell suspension was filtered through a 40-μm nylon mesh, after which it was processed for antibody staining and flow cytometry.

### Flow cytometry analysis of mouse tissue

Flow cytometry measurements were obtained using an LSRFortessa X-20 with the following setup: UV laser (355 nm) with 379/28 and 525/50 filters; violet laser (405 nm) with 450/50, 525/50, 610/20, 670/80, 710/50 and 780/60 filters; blue laser (488 nm) with 530/30 and 710/50 filters; yellow/green laser (561 nm) with 586/15, 610/20, 670/30, 710/50 and 780/60 filters; and red (640 nm) laser with 670/30, 730/45 and 780/60 filters. Data analysis was performed with FlowJo version 10.

#### Peripheral blood

After washing of peripheral leukocytes, cells were stained with antibodies against CD45 (BioLegend, clone 30-F11), CD11b (BioLegend, clone M1/70), CD90 (BioLegend, clone 30-H12), Ly6G (BioLegend, clone 1A8), Cd115 (BioLegend, clone AFS98), CX3CR1 (BioLegend, clone SA011F11), CD19 (BD Biosciences, clone 1D3) and Ly6C (BioLegend, clone HK1.4). Immune cell subsets were identified as follows: monocytes (CD45^high^, CD11b^high^, Ly6G^lo^^w^, CD115^high^, CX3CR1^high^, Ly6C^low^^/high^), neutrophils (CD45^high^, CD11b^high^, Ly6G^high^), T cells (CD45^high^ CD11b^low^, Cd90^high^) and B cells (CD45^high^, CD11b^low^, CD19^high^).

#### Heart

Single-cell suspensions of mouse hearts were stained with antibodies against CD45 (BioLegend, clone 30-F11), Ly6C (BioLegend, clone HK1.4), F4/80 (BioLegend, clone BM8), CD11b (BioLegend, clone M1/70), Ly6G (BioLegend, clone RB6-8C5), CD90 (BioLegend, clone 30-H12), CD19 (BD Biosciences, clone 1D3) and CCR2 (BioLegend, clone SA203G11). Cardiac immune cells were defined as follows: monocytes (CD45^high^, Ly6G^low^, Ly6C^high^, F4/80^low^), macrophages (CD45^high^, Ly6G^low^, Ly6C^low^, F4/80^high^), recruited macrophages (CD45^high^, Ly6G^low^, Ly6C^low^, F4/80^high^, CCR2^+^), neutrophils (CD45^high^, CD11b^high^, Ly6G^high^), T cells (CD45^high^ CD11b^low^, CD90^high^) and B cells (CD45^high^, CD11b^low^, CD19^high^).

#### Bone marrow

After isolation of bone marrow, single-cell suspensions were initially stained with the following biotin-conjugated antibodies for lineage exclusion: CD3 (BioLegend, clone 145-2C11), CD4 (BioLegend, clone GK1.5), CD8 (BioLegend, clone 53-6.7), CD90.2 (BioLegend, clone 30-H12), CD19 (BioLegend, clone 6D5), B220 (BioLegend, clone RA3-6B2), NK1.1 (BioLegend, clone PK136), Ter119 (BioLegend, clone TER-119), CD11b (BioLegend, clone M170), CD11c (BioLegend, clone N418) and Gr1 (BioLegend, clone RB6-8C5). After lineage exclusion, hematopoietic progenitors and Lin^+^ cells were stained with the following antibodies: streptavidin (BioLegend), CD48 (BioLegend, clone HM48-1), c-kit (BioLegend, clone QA17A09), CD16/32 (BioLegend, clone 93), Flt3 (BioLegend, clone A2F10), CD34 (BioLegend, clone RAM34), Sca1 (BioLegend, clone D7) and CD150 (BioLegend, clone TC15-12F12.2). Bone marrow progenitor populations were defined as follows: LKs (Lin^−^c-kit^+^Sca1^−^), LSKs (Lin^−^c-kit^+^Sca1^+^), GMPs (Lin^−^c-kit^+^Sca1^−^CD34^+^CD16/32^+^) and LT-HSCs (Lin^−^c-kit^+^Sca1^+^Flt3^−^CD48^−^CD150^+^).

### Mouse tissue histology and immunofluorescent staining

Hearts were fixed in 4% paraformaldehyde (PFA) and subsequently embedded in paraffin. The tissue was then sectioned into 4-μm-thick slices using a microtome. After deparaffinization, the slides were stained with picrosirius red to assess fibrosis. The slides were mounted with ProLong Gold Antifade Mountant (Thermo Fisher Scientific), and images were captured with a Nikon Eclipse Ti2 microscope. For immunofluorescent staining of macrophages in the post-MI heart, a primary rabbit anti-mouse CD68 antibody (Cell Signaling Technology, clone E307V) was used, along with a secondary polyclonal fluorochrome-conjugated donkey anti-rabbit antibody (Thermo Fisher Scientific). The slides were mounted with ProLong Gold Antifade Mountant (Thermo Fisher Scientific), and imaging was performed on a Zeiss LSM 780 Observer Z.1 microscope. Image analysis was carried out using ZEN software and ImageJ. CD68^+^ cell density was calculated as the number of CD68^+^ cells per region of interest (ROI), normalized to area (cells per mm^2^), where each ROI was 212.55 × 212.55 µm.

### RNA isolation, cDNA synthesis and quantitative RT−qPCR

Total RNA was isolated using the RNeasy Plus Mini Kit (Qiagen) following the manufacturer’s guidelines. RNA content and purity were assessed by spectrophotometry (NanoDrop Technologies). mRNA expression was quantified by RT−qPCR using total RNA, which was reversed transcribed by MuLV reverse transcriptase (Thermo Fisher Scientific) and random hexamer primers (Thermo Fisher Scientific). cDNA synthesis was performed according to the following protocol: (primer annealing) 65 °C × 5 minutes, (primer elongation) 25 °C × 10 minutes, (reverse transcription) 37 °C × 50 minutes and (enzyme denaturation) 70°C × 15 minues. Expression levels of mRNA were detected by using Fast SYBR Green (Applied Biosystems) and an Applied Biosystems ViiA7 machine. Cycling conditions on the machine were as follows: (hold) 95 °C × 30 seconds, (PCR) 95 °C × 1 seconds, 60 °C × 20 seconds repeated for 40 cycles and (melt curve) 95 °C × 15 seconds, 60 °C × 60 seconds and 95 °C × 15 seconds. Expression levels were additionally detected using the TaqMan-based system using TaqMan Fast Advanced Master Mix. Cycling conditions on the machine were as follows: (hold) 95 °C × 20 seconds and (PCR) 95 °C × 1 seconds, 60 °C × 20 seconds repeated for 40 cycles. Relative gene expression was calculated with QuantStudio Real-Time PCR software (version 1.3) using ${2}^{-{\Delta}{C}_{T}}$ or ${2}^{-{\Delta\Delta}{C}_{T}}$. *Rplp0* was used as housekeeping gene. SYBR Green sequences and TaqMan probe IDs can be found in Supplementary Table 1.

### ELISA on mouse plasma

Mouse plasma was via centrifugation of peripheral blood at 1,500*g* (4 °C, 15 minutes), after which the supernanant (plasma) was transferred to a new tube for further analysis. Analytes were detected using a Mouse Luminex Discovery Assay (Bio-Techne) according to the manufacturer’s instructions. Analytes were measured using a Bio-Plex 100 (Bio-Rad).

### Cardiac CD45^+^ cell isolation for scRNA-seq

Heart tissue processing was performed according to the ‘Mouse tissue processing’ with a few alterations. After obtaining the cardiac single-cell suspension, a tissue debris removal was used according to the manufacturer’s instructions (Miltenyi Biotec). Subsequently, red blood cells were lysed with 1× RBC lysis buffer (BioLegend) for 5 minutes. After washing, cells were stained with CD45 (BioLegend, clone 30-F11) and 7-AAD (BD Biosciences), and CD45^high^7-AAD^−^ cells were sorted for downstream single-cell analyses. Sorting was performed with a FACSAria Fusion (BD Biosciences) with the following setup: UV laser (355 nm) with 379/28 and 730/45 filters; violet laser (405 nm) with 450/40, 525/50, 610/20, 660/20, 710/50 and 780/60 filters; blue laser (488 nm) with 488/10, 530/30, 584/42, 616/23, 695/40 and 780/60 filters; and red laser (640 nm) with 670/30, 730/45 and 780/60 filters.

### Nuclei isolation from cardiac CD45^+^ cells for scATAC−seq

Mouse cardiac tissue processing for CD45^+^ cell sorting was processed as described in the ‘Cardiac CD45+ cell isolation for scRNA-seq’. Nuclei were extracted with a modified version of the 10x Genomics ‘Nuclei Isolation for Single Cell ATAC Sequencing’ protocol. In brief, CD45^+^ cells were centrifuged (300*g*, 5 minutes, 4 °C) and resuspended in pre-chilled lysis solution (Tris-HCl, pH 7.4, 10 mM, NaCl 10 mM, MgCl_2_ 3 mM, 0.1% Tween 20, 0.1% Nonidet P40 Substitute, 0.01% digitonin, 1% BSA) for 5 minutes on ice. Nuclei were then washed with a 1-ml wash solution (Tris-HCl, pH 7.4, 10 mM, NaCl 10 mM, MgCl_2_ 3 mM, 0.1% Tween 20, 1% BSA) and centrifuged (500*g*, 5 minutes, 4 °C). Viable nuclei were stained with 7-AAD, and 7-AAD^high^ nuclei were FACS sorted before proceeding to downstream scATAC library preparation. Sorting was performed with a FACSAria Fusion (BD Biosciences) (setup described in the ‘Cardiac CD45+ cell isolation for scRNA-seq’).

### Single-cell RNA-seq

Viable mouse CD45^+^ cell suspensions or human PBMCs were processed according to the manufacturer’s instructions, with cells loaded onto a 10x Chromium Controller (10x Genomics). Human patient samples were screened for additional CH driver mutations. Patient characteristics can be found in Supplementary Table 1. No co-occurring CH driver mutations were detected, ensuring that the patient group used for downstream analyses represented KDM6A-driven CH without detectable additional CH driver events. The libraries for scRNA-seq were prepared using the Chromium Single Cell 3′ v3 Reagent Kit (10x Genomics). Initially, individual cells were isolated into droplets within an emulsion, each containing gel beads coated with unique primers that carried 10x cell barcodes, unique molecular identifiers and poly(dT) sequences. Reverse transcription then generated barcoded full-length cDNA, which was followed by emulsion disruption using a recovery agent and cDNA purification with DynaBeads MyOne Silane Beads (Thermo Fisher Scientific). Amplification of cDNA was performed on a Biometra Thermocycler TProfessional Basic Gradient with a 96-well sample block under the following conditions: 98 °C for 3 minutes, followed by 14 cycles of 98 °C for 15 seconds, 67 °C for 20 seconds and 72 °C for 1 minute, ending with a 72 °C step for 1 minute and holding at 4 °C. The amplified cDNA product was purified using the SPRIselect Reagent Kit (Beckman Coulter). To construct the indexed sequencing libraries, the Chromium Single Cell 3′ v3 Reagent Kit was used to carry out fragmentation, end repair, A-tailing and size selection with SPRIselect. Additional steps included adaptor ligation, post-ligation cleanup with SPRIselect, PCR for sample index and cleanup using SPRIselect beads. Quantification and quality assessment of the libraries were done using a Bioanalyzer 2100 (Agilent Technologies) with a High-Sensitivity DNA chip (Agilent Technologies). The indexed libraries were pooled equimolarly and sequenced on two Illumina NovaSeq 6000 instruments with paired-end sequencing (26 × 98 bp) at GenomeScan BV.

### Single-cell RNA-seq data analysis

FASTQ files were mapped to the human (GRCh38) or the mouse (mm10) genome using Cell Ranger 7.1 (10x Genomics). Filtered matrices were analyzed using Seurat 5.2 (ref. ^51^) in RStudio with R 4.4.2. Quality control steps included removing features detected in fewer than three cells and removing cells with fewer than 200 features, more than 6,000 genes and more than 20% mitochondrial RNA. Data were normalized by scaling feature expression by the total expression per cell, multiplied by a scale factor of 10,000 and then log transformed. This was followed by selection of features that exhibit high cell-to-cell variation, and data were linearly transformed prior to dimensionality reduction. Data were subjected to principal component analysis and unsupervised clustering by the Louvain clustering method. The number of principal components to apply was determined using an elbow plot in Seurat. Samples were integrated using canonical correlation analysis. Cell clusters were visualized using UMAP and were manually annotated using published markers of immune cell subsets. Genes specific to or within each cluster for *Kdm6a*^WT^ versus *Kdm6a*^*Δ-Haem*^ comparison were generated using the FindMarkers function. Gene Ontology analysis was performed using the clusterProfiler package (version 4.14.4). Volcano plots were generated using EnhancedVolcano in R (version 1.24.0). UpSet plots were generated with UpSetR (version 1.4.0).

### Single-cell ATAC−seq

The nuclei suspensions were counted manually and diluted according to the manufacturer’s protocol to obtain 10,000 single-cell data points per sample. Each sample was run separately on a lane in a Chromium Controller with Chromium Next GEM Single Cell ATAC Reagent Kits version 1.1 (10x Genomics). Single-cell ATAC−seq library preparation was done using standard protocol. Sequencing was done on NextSeq 2000.

### Single-cell ATAC−seq data analysis

Raw reads were aligned against the mouse genome (mm39, Ensembl 104 release) and counted by STARsolo^52^. Peak calling was done by MACS^53^, and the parameters used were as follows: --nomodel --shift -100 --extsize 200 --broad. Preprocessed counts were further analyzed using Scanpy^54^. Basic cell quality control was conducted by the following criteria: (1) mean fragment length = 300, (2) 7.8 < total counts per cell (log) < 12, (3) reads in peaks > 0.2 and (4) reads in Chr.M < 0.1. Downstream analyses of peak versus cells matrices were performed using Signac^55^ (1.14.0) in RStudio with R 4.4.2. In brief, data were normalized via term frequency−inverse document frequency (TF-IDF) normalization. Top variable features were selected for dimensional reduction with singular value decomposition (SVD) on the TD-IDF matrix for latent semantic indexing (LSI). The first LSI component was removed from downstream analysis as it was correlated with sequencing depth (visualized with the DepthCor() function in Signac). Cell clusters were then visualized in a low-dimensional space with UMAP. A gene activity matrix was computed by summing the fragments intersecting the gene body and promoter region to visualize canonical markers and interpret cell clusters and subsequently log normalized. To additionally help with the deconvolution of cell clusters, we used our unpaired scRNA-seq data to identify correlation patterns between the gene activity matrix and scRNA-seq data. This was achieved by identifying a set of anchors between the RNA and ATAC data, followed by a transfer of cell labels to the ATAC data. Gene activity was then visualized with CoveragePlot() to ensure validity of cell type assignment. Peaks specific to or within each cluster for *Kdm6a*^WT^ versus *Kdm6a*^*Δ-Haem*^ comparison were generated using the FindMarkers function. ATAC peaks were linked to genes for each cell cluster with the gABC-scoring function from STARE (version 1.0.4)^56^. The unified ATAC peaks were used as candidate enhancers, and the counts per million (CPM) in each peak of each cell cluster was used as enhancer activity. Scanpy was used for file access^54^. As gene annotation, the M27 version from GENCODE was used^57^. The window size for STARE was set to 5 megabases and the score cutoff to 0.02, and, as contact, the estimate based on distance was chosen as follows: ‘STARE_ABCpp -a gencode.vM27.annotation.gtf.gz -b <unified ATAC peaks > -n 5 + -w 5000000 -f False -o <output_dir>’. Processing involving genomic coordinates made use of pybedtools (0.8.1)^58^. For analysis of the signal coverage, the BAM files for each cell cluster were created with the help of the bam-merge-master functionality from STITCHIT^59^ and subsequently converted to BigWig files with bamCoverage from deepTools (version 3.5.1) (normalize with CPM and bin size of 20)^60^. The coverage plots were then generated with the plotHeatmap function from deepTools. For determining the location of regions with respect to genes, each base pair was assigned to a location and the percentage given in relation to the summed base pairs of all peaks. Promoters were defined as ±200 bp around all annotated transcription start sites of all genes, and introns were defined as parts of the gene body that were not labeled as anything else. Colors for the locations were taken from the colorcet Python package based on Glasbey et al.^61^. Volcano plots were generated using EnhancedVolcano in R (version 1.24.0). UpSet plots were generated with UpSetR (version 1.4.0).

### Bulk mRNA sequencing on mouse heart tissue

For genome-wide analysis of gene expression, RNA-seq libraries from isolated mRNA were generated and sequenced by the Institute for Lung Health, Genomics and Bioinformatics, at the Justus Liebig University Giessen. Total RNA (200 ng) was used to enrich for polyadenylated mRNA followed by cDNA sequencing library preparation utilizing the Illumina Stranded mRNA Prep Kit (Illumina) according to the manufacturer’s instructions. After library quality control by capillary electrophoresis (Agilent Technologies, 4200 TapeStation), cDNA libraries were sequenced on the Illumina NextSeq 2000 platform generating 50-bp paired-end reads. Reads were mapped via STAR (version 2.7.11b) against the mouse reference genome (GRCM38.97) with the additional parameters --quantMode GeneCounts and --outSAMtype BAM SortedByCoordinate. Gene count matrices were analyzed with DESeq2 (version 1.52.0)^62^ in R. Differential gene expression was performed comparing anti-IL-1β-treated samples with IgG controls with treatment specified as the design factor. Lowly expressed genes were filtered by retaining genes with counts ≥10 in at least two samples. Gene set enrichment analysis was performed with fgsea with mouse Molecular Signatures Database (MSigDB) Hallmark gene sets. Genes were ranked by the DESeq2 Wald statistic, and enrichment significance was assessed using false discovery rate (FDR)-adjusted *P* values.

### Transcription factor footprinting analysis with TOBIAS

For the transcription factor footprinting analysis, the TOBIAS tool was utilized^63^. The scATAC data were preprocessed by the scframework^64^ derived standardized Jupyter notebooks, and cells were grouped according to their cell type. These were then separated by condition (WT/KO) to cell barcodes, and the data were summarzed as pseudobulks from the global BAM file in order to cope with the sparseness of single cells. Per cell type, the two pseudobulk BAM files representing the conditions were fed to TOBIAS by making use of the TOBIAS snakemake pipeline. For processing, the following were used: ensemble mm10 release 104 blacklisted regions, fasta sequences and respective gtf file, as well as the JASPAR core vertebrate database from 2024 (10th release)^65^.

### Epigenetic LISA

Differentially expressed genes identified in the mouse cardiac CD45^+^ scRNA-seq data were uploaded to LISA (http://lisa.cistrome.org/) to identify upstream chromatin regulators of differentially expressed genes. Output consisted of adjusted *P* values. Inferred chromatin regulators data for CCR2^+^ macrophages and neutrophils were visualized according to significance in Extended Data Fig. 6.

### Integration of scRNA-seq/snRNA-seq and cell communication analyses

Integration of scRNA-seq and snRNA-seq data for cell−cell communication analyses was performed as previously described^14^. In brief, we filtered monocytes via the subset() function of whole PBMC dataset from patients with HF with no CH and patients with HF with KDM6A CH. To integrate snRNA-seq data of cardiac tissue and scRNA-seq datasets of PBMCs, we followed the integration as described in the ‘Single-cell RNA-seq data analysis’ with some alterations. To improve integration of data, we utilized Harmony^66^ with sample identity and sequencing technology specified as batch covariates. For crosstalk analysis, we used CellChat (2.1.2 version)^12^. We followed the standard tutorial ‘Comparison analysis of multiple datasets using CellChat’ from the CellChat GitHub repository (https://github.com/jinworks/CellChat).

### PBMC isolation and monocyte-to-macrophage differentiation

For in vitro KDM6A silencing experiments, PBMCs were isolated from buffy coats of healthy donors, obtained from Blutspendedienst Frankfurt, by density gradient centrifugation. In brief, PBMC-enriched blood was diluted 1:3 with pre-warmed PBS and layered on top of human Pancoll (1.077 g ml^−1^; PAN Biotech), followed by centrifugation at 800*g* for 20 minutes at room temperature without brakes applied. The white ring of PBMCs was transferred to a new 50-ml Falcon tube, and PBS was added to bring the total volume to 50 ml. The sample was then centrifuged at 300*g* for 10 minutes at room temperature, with acceleration and brakes enabled. The supernatant was discarded, and the pellet was resuspended in 50 ml of PBS. Platelets were removed by two additional washing steps and centrifugation at 200*g* for 10 minutes, and the supernatant was discarded. CD14^+^ monocytes were isolated using magnetic-activated cell sorting (Miltenyi Biotec) with CD14 MicroBeads UltraPure (Miltenyi Biotec) before proceeding to monocyte-to-macrophage differentiation. Monocytes were differentiated to macrophages with RPMI 1640 media supplemented with 10% FBS, 1% penicillin−streptomycin, 10 mM HEPES, 1 mM sodium pyruvate and 20 ng ml^−1^ macrophage colony-stimulating factor (M-CSF) for 7 days, with medium change every second day, before proceeding to downstream assays. Donors of buffy coats were three healthy aged males (65−70 years), with no loss of Y chromosome in all three patients detected by digital PCR. One of the donors carried a *DNMT3A* splice site mutation (variant allele frequency (VAF) 5.70%); another donor carried a *TET2* mutation (VAF 2.22%); and the third donor did not show mutations in *DNMT3A* and *TET2*, detected in collaboration with Münchner Leukämielabor.

### Small interfering RNA knockdown of KDM6A in CD14^+^ monocyte-derived macrophages

KDM6A was silenced in CD14^+^ monocyte-derived macrophages with 100 nM ON-TARGETplus Human KDM6A siRNA SMARTPool (Horizon Dharmacon) by using siTran2.0 transfection reagent (OriGene) and 100 nM ON-TARGETplus siRNA Non-targeting Pool as siRNA control (Horizon Dharmacon). In brief, the medium was replaced with 1 ml of fresh complete medium 30 minutes before transfection. Subsequently, the transfection mix was prepared by adding components in the order specified and vortexing the mixture as follows: 92.1 µl of 1× transfection buffer, 5.5 µl of 20 µM siRNA and 2.4 µl of siTran 2.0 siRNA transfection reagent. The mixture was then incubated for 20 minutes. After incubation, the transfection mix was added onto the cells in a dropwise manner, and the plate was moved back and forth to evenly distribute the mix. The media were refreshed 6 hours after transfection. Finally, supernatants from transfected cells were collected 48 hours after transfection, and the cells were analyzed by qPCR.

### Human cardiac fibroblast activation assay

Primary human cardiac fibroblasts (HCF-c) (PromoCell, C-12375) were cultured in Fibroblast Growth Medium 3 (PromoCell) according to the manufacturer’s protocol. HCF-c were seeded at a density of 1,200 cells per well in 18-well µ-Slides (ibidi) coated with human fibronectin (Sigma-Aldrich, 0.1% solution, 1:200 in water). The next day, in indirect co-culture experiments, fibroblasts were incubated with supernatants of transfected CD14^+^ monocyte-derived macrophages. The supernatants were collected 48 hours after transfection and stored at −80 °C. Macrophage supernatant was mixed with the fibroblast cell medium at a ratio of 1:2 (co-culture medium, 20 µl of supernatant and 40 µl of medium). Fibroblasts were incubated with this co-culture medium for 72 hours, fixed with 4% PFA (in DPBS; Thermo Fisher Scientific) and then used for immunofluorescent staining.

### Neonatal rat cardiomyocyte stress assay—beating frequency and hypertrophy measurements

Neonatal rat cardiomyocytes (NRCMs) were isolated from Sprague Dawley postnatal day 1 and postnatal day 2 rat pups. In brief, pregnant female Sprague Dawley rats were obtained from Janvier Labs. Rat pups were euthanized by cervical dislocation, and hearts were transferred into HBSS (−Ca^2+^/−Mg^2+^; Gibco) containing 0.2% 2,3-butanedione monoxime (BDM; Sigma-Aldrich). Hearts were cut in small pieces and dissociated at 37 °C with the enzyme mix (Neonatal Heart Dissociation Kit, mouse and rat; Miltenyi Biotec), followed by tissue homogenization with the gentleMACS Dissociator (Miltenyi Biotec; program m_neoheart_01_01) in a C-tube (Miltenyi Biotec). Cells were transferred to a 70-µm strainer (Falcon), centrifuged (80*g*, 5 minutes) and resuspended in plating medium (DMEM high glucose and M199 EBS, both from BioConcept, supplemented with 10% horse serum from Thermo Fisher Scientific), 5% fetal calf serum (Gibco), 2% L-glutamine (Thermo Fisher Scientific) and penicillin−streptomycin (Roche) and incubated for 1 hour, 40 minutes in 10-cm cell culture dishes (Greiner Bio-One) at 37 °C and 5% CO_2_. Within the incubation time, the fibroblasts attach to the uncoated culture dish, and the supernatant contains the cardiomyocytes and was collected. Cells were counted with a Neubauer chamber (Carl Roth).

NRCMs were seeded in plating medium at a density of 5,000 cells per well in 18-well µ-Slides (ibidi) coated with rat tail collagen type I (Corning; 1:200 in water). One day after isolation, the medium of cultured NRCMs was changed to maintenance medium (DMEM high glucose and M199 EBS, both without L-glutamine (BioConcept)), 1% horse serum, 2% L-glutamine and penicillin−streptomycin. For indirect co-culture experiments, NRCMs were incubated with supernatants of transfected CD14^+^ monocyte-derived macrophages. Macrophage supernatant was mixed with the maintanance medium at a ratio of 1:2 (co-culture medium, 20 µl of supernatant and 40 µl of medium). NRCMs were incubated with this co-culture medium for 48 hours (beating frequency measurement) and for 72 hours (hypertrophy assay).

The cardiomyocyte beating frequency was determined 48 hours after NRCM plating by using bright-field microscope videos (Zeiss) and counting the number of beats per minute. For hypertrophic cell size assessment, cardiomyocytes were fixed with 4% PFA (in DPBS; Thermo Fisher Scientific) for immunofluorescence staining.

### Immunofluorescence staining for cell culture

After indirect co-culture, cells were washed two times with DBPS and fixed with 4% PFA (Thermo Fisher Scientific) for 10 minutes at room temperature. After two washing steps, each for 5 minutes, with DPBS, cells were permeabilized with 0.1% Triton X-100 (Thermo Fisher Scientific) in DPBS for 10 minutes. Cells were blocked with 5% donkey serum (Abcam) in DPBS for 60 minutes at room temperature. Primary antibodies were incubated in the same blocking solution overnight at 4 °C. HCF-c were stained with phalloidin (1:400; Thermo Fisher Scientific) and rabbit anti-collagen type 1A1 (1:200; Cell Signaling Technology). NRCMs were stained with phalloidin (1:400; Thermo Fisher Scientific) and mouse anti-α-actinin (sarcomeric) antibody (1:200; Thermo Fisher Scientific). Cells were washed four times with DPBS, each 5 minutes, and secondary antibodies in DPBS were incubated for 1 hour at room temperature. The following dyes and secondary antibodies were used: for HCF-c: DAPI (1:1,000; Merck) and donkey anti-rabbit-647 (1:200; Thermo Fisher Scientific); for NRCMs: DAPI (1:1,000; Merck) and donkey anti-mouse-647 (1:200; Thermo Fisher Scientific). Cells were mounted with Fluoromount-G (Invitrogen). Images were taken using a Leica STELLARIS confocal microscope. Then, *z*-stack images were acquired with a resolution of 1,024 × 1,024 pixels, a speed of 600 frames per second, line average 2 and a *z*-stack size of approximately 1 µm. Quantification was done using Volocity software version 6.5 (Quorum Technologies).

### Statistical analysis

Statistical analyses were performed using GraphPad Prism 10 (GraphPad Software). When Shapiro−Wilk normality test indicated a Gaussian distribution, an unpaired *t*-test was used, as indicated. Otherwise, Mann−Whitney tests were used. Data are represented as mean ± s.e.m., and *P* < 0.05 was considered significant. For single-cell sequencing RNA experiments, a two-tailed Wilcoxon rank-sum test with subsequent Bonferroni correction was used to identify differential genes. For single-cell ATAC experiments, a logistic regression test with subsequent Bonferroni correction was used to identify differential peaks. For the anti-IL-1β in vivo intervention experiment, the timecourse EF data were analyzed using two-way repeated-measures ANOVA with treatment and time as factors, followed by Sidak’s multiple-comparisons test to compare groups within each timepoint.

### Reporting summary

Further information on research design is available in the Nature Portfolio Reporting Summary linked to this article.

## Supplementary information

Reporting Summary

Supplementary Table 1. One Excel file with four labeled tabs: (1) Genotyping primers; (2) qPCR primers and TaqMan probes; (3) Human patient clinical characteristics; (4) RNA sequencing intersection of genes.

## Source data

Source Data Figs. 1, 5 and 6 and Extended Data Figs. 1−3, 5 and 10 Raw data.

## Data Availability

Sequencing data supporting the findings of this study have been deposited in the ArrayExpress data depository with the following accession numbers: mouse scRNA-seq data (E-MTAB-17561), mouse scATAC−seq data (E-MTAB-17569), mouse bulk RNA-seq data (E-MTAB-17560) and human scRNA-seq data (E-MTAB-17614). Additional data in this study are included as source data.
