# Supplementary material for: Hematopoietic loss of KDM6A impairs cardiac recovery in heart failure via epigenetic reprogramming of myeloid cells
Source: Nat Cardiovasc Res. 2026 Nov 3;5(11):1193–212. doi: 10.1038/s44161-026-00887-3 (PMC13759310; doi:10.1038/s44161-026-00887-3)
Supplement: Supplementary file 1 [file 44161_2026_887_MOESM1_ESM.pdf]

nature portfolio

Corresponding author(s): Sebastian Cremer

Last updated by author(s): Sep 4, 2026

Reporting Summary

Nature Portfolio wishes to improve the reproducibility of the work that we publish. This form provides structure for consistency and transparency in reporting. For further information on Nature Portfolio policies, see our Editorial Policies and the Editorial Policy Checklist.

Statistics

For all statistical analyses, confirm that the following items are present in the figure legend, table legend, main text, or Methods section.

n/a	Confirmed	
☐	☒	The exact sample size (*n*) for each experimental group/condition, given as a discrete number and unit of measurement
☐	☒	A statement on whether measurements were taken from distinct samples or whether the same sample was measured repeatedly
☐	☒	The statistical test(s) used AND whether they are one- or two-sided<br>*Only common tests should be described solely by name; describe more complex techniques in the Methods section.*
☐	☒	A description of all covariates tested
☐	☒	A description of any assumptions or corrections, such as tests of normality and adjustment for multiple comparisons
☐	☒	A full description of the statistical parameters including central tendency (e.g. means) or other basic estimates (e.g. regression coefficient) AND variation (e.g. standard deviation) or associated estimates of uncertainty (e.g. confidence intervals)
☐	☒	For null hypothesis testing, the test statistic (e.g. *F*, *t*, *r*) with confidence intervals, effect sizes, degrees of freedom and *P* value noted<br>*Give P values as exact values whenever suitable.*
☒	☐	For Bayesian analysis, information on the choice of priors and Markov chain Monte Carlo settings
☒	☐	For hierarchical and complex designs, identification of the appropriate level for tests and full reporting of outcomes
☒	☐	Estimates of effect sizes (e.g. Cohen's *d*, Pearson's *r*), indicating how they were calculated

Our web collection on statistics for biologists contains articles on many of the points above.

Software and code

Policy information about availability of computer code

Data collection	Vevo 3100, LSRFortessa X-20, Aria Fusion, 780 Zeiss confocal microscope, Nikon Eclipse Ti2 microscope, Applied Biosystems Biosystems Viia7, 10x Genomics Chromium, Illumina Novaseq 6000, Nextseq 2000
Data analysis	Flowjow v10, VevoLab 5.5.0, ImageJ/Fiji, Graphpad Prism, Cellranger v7, StarSolo, MACS, R, Python, TOBIAS, LISA, BASH, CellChat, Volocity 6.5

For manuscripts utilizing custom algorithms or software that are central to the research but not yet described in published literature, software must be made available to editors and reviewers. We strongly encourage code deposition in a community repository (e.g. GitHub). See the Nature Portfolio guidelines for submitting code & software for further information.

Data

Policy information about availability of data

All manuscripts must include a data availability statement. This statement should provide the following information, where applicable:

- Accession codes, unique identifiers, or web links for publicly available datasets
- A description of any restrictions on data availability
- For clinical datasets or third party data, please ensure that the statement adheres to our policy

Sequencing data supporting the findings of this study have been deposited in the ArrayExpress Data Depository with the following accession numbers: mouse single-cell RNA sequencing data (E-MTAB-17561), mouse single-cell ATAC sequencing data (E-MTAB-17569), mouse bulk RNA sequencing data (E-MTAB-17560), human single-cell RNA sequencing data (E-MTAB-17614). Additional data in this study are included as source data.

Research involving human participants, their data, or biological material

Policy information about studies with human participants or human data. See also policy information about sex, gender (identity/presentation), and sexual orientation and race, ethnicity and racism.

Reporting on sex and gender	Characteristics of human samples used for single cell RNA sequencing can be found in the Supplementary Tables. Human samples used for in-vitro experiments can be found under Method section "Peripheral Blood Mononuclear Cells (PBMCs) isolation and monocyte to macrophage differentiation".
Reporting on race, ethnicity, or other socially relevant groupings	Our study does not report on the race or ethnicity of participants.
Population characteristics	Information can be found in Supplementary Tables and under Method section "Peripheral Blood Mononuclear Cells (PBMCs) isolation and monocyte to macrophage differentiation"
Recruitment	Partcipants were recruited based on data bank status and willingness to participate.
Ethics oversight	Ethics committee University Hospital Frankfurt

Note that full information on the approval of the study protocol must also be provided in the manuscript.

Field-specific reporting

Please select the one below that is the best fit for your research. If you are not sure, read the appropriate sections before making your selection.

☒ Life sciences ☐ Behavioural & social sciences ☐ Ecological, evolutionary & environmental sciences

For a reference copy of the document with all sections, see nature.com/documents/nr-reporting-summary-flat.pdf

Life sciences study design

All studies must disclose on these points even when the disclosure is negative.

Sample size	Sample size was determined based on prior experience. Additionally, sample sizes were calculated with power=0.8, type I error = 0.05 .
Data exclusions	No data was excluded
Replication	When possible experiments were replicated at least 3 times.
Randomization	Randomisation of human samples was done when possible.
Blinding	Investigators were blinded during data analysis.

Reporting for specific materials, systems and methods

We require information from authors about some types of materials, experimental systems and methods used in many studies. Here, indicate whether each material, system or method listed is relevant to your study. If you are not sure if a list item applies to your research, read the appropriate section before selecting a response.

Materials & experimental systems

n/a	Involved in the study	
☐	☒	Antibodies
☐	☒	Eukaryotic cell lines
☒	☐	Palaeontology and archaeology
☐	☒	Animals and other organisms
☒	☐	Clinical data
☒	☐	Dual use research of concern
☒	☐	Plants

Methods

n/a	Involved in the study	
☒	☐	ChIP-seq
☐	☒	Flow cytometry
☒	☐	MRI-based neuroimaging

Antibodies

Antibodies used	Peripheral blood: Following washing of peripheral leukocytes, cells were stained with antibodies against CD45 (Biolegend, clone 30-F11), CD11b (Biolegend, clone M1/70), CD90 (Biolegend, clone 30-H12), Ly6G (Biolegend, clone 1A8), Cd115 (Biolegend, clone

	AFS98), CX3CR1 (Biolegend, clone SA011F11), CD19 (BD Bioscience, clone 1D3) and Ly6C (Biolegend, clone HK1.4).<br>Heart: Single-cell suspensions of murine hearts were stained with antibodies against CD45 (Biolegend, clone 30-F11), Ly6C (Biolegend, clone HK1.4), F4/80 (Biolegend, clone BM8), CD11b (Biolegend, clone M1/70) , Ly6G (Biolegend, clone RB6-8C5), CD90 (Biolegend, clone 30-H12), CD19 (BD Bioscience, clone 1D3), CCR2 (Biolegend, clone SA203G11<br>Bone marrow: Following isolation of bone marrow, single cell suspensions were initially stained with the following biotin-conjugated antibodies for lineage exclusion: CD3 (Biolegend, clone 145-2C11, CD4 (Biolegend, clone GK1.5, CD8 (Biolegend, clone 53-6.7, CD90.2 (Biolegend, clone 30-H12, CD19 (Biolegend, clone 6D5, B220 (Biolegend, clone RA3-6B2, NK1.1 (Biolegend, clone PK136 ,Ter119 (Biolegend, clone TER-119, CD11b (Biolegend, clone M170, CD11c (Biolegend, clone N418, Gr1 (Biolegend, clone RB6-8C5). Following lineage exclusion, haematopoietic progenitors and lineage+ cells were stained with the following antibodies: Streptavidin (Biolegend), CD48 (Biolegend, clone HM48-1), c-kit (Biolegend, clone QA17A09), CD16/32 (Biolegend, clone 93), Flt3 (Biolegend, clone A2F10), CD34 (Biolegend, clone RAM34), Sca1(Biolegend, clone D7), CD150 (Biolegend, clone TC15-12F12.2)
Validation	Validation was done by corresponding companies.

Eukaryotic cell lines

Policy information about cell lines and Sex and Gender in Research

Cell line source(s)	Primary human cardiac fibroblasts (HCF-c) (#C-12375, Promocell)
Authentication	Authentication not done in-house but from the company (Promocell)
Mycoplasma contamination	Testing done from company (Promocell)
Commonly misidentified lines (See ICLAC register)	*Name any commonly misidentified cell lines used in the study and provide a rationale for their use.*

Animals and other research organisms

Policy information about studies involving animals; ARRIVE guidelines recommended for reporting animal research, and Sex and Gender in Research

Laboratory animals	VaviCre (B6.Cg-Commd10Tg(Vav1-icre)A2Kio/J, Jackson laboratory) heterozygous male mice were bred with female Kdm6afl/fl (B6;129S-Kdm6atm1.1Kaig/J, Jackson laboratory) homozygous mice to establish VaviCreKdm6afl/fl offspring with a haematopoietic specific KDM6A knockout.<br><br>Neonatal rat cardiomyocytes (NRCM) were isolated from Sprague Dawley P1 and P2 rat pups. Pregnant female Sprague Dawley rats were obtained from Janvier (Le Genest Saint-Isle, France).
Wild animals	No wild animals were used
Reporting on sex	Sex of animals is reported in-text, in corresponding figures and in Methods section.
Field-collected samples	This study does not involve fileld-collected samples
Ethics oversight	The studies were approved by the local ethics committee (Regierungspräsidium Darmstadt, Hessen)

Note that full information on the approval of the study protocol must also be provided in the manuscript.

Plants

Seed stocks	*Report on the source of all seed stocks or other plant material used. If applicable, state the seed stock centre and catalogue number. If plant specimens were collected from the field, describe the collection location, date and sampling procedures.*
Novel plant genotypes	*Describe the methods by which all novel plant genotypes were produced. This includes those generated by transgenic approaches, gene editing, chemical/radiation-based mutagenesis and hybridization. For transgenic lines, describe the transformation method, the number of independent lines analyzed and the generation upon which experiments were performed. For gene-edited lines, describe the editor used, the endogenous sequence targeted for editing, the targeting guide RNA sequence (if applicable) and how the editor was applied.*
Authentication	*Describe any authentication procedures for each seed stock used or novel genotype generated. Describe any experiments used to assess the effect of a mutation and, where applicable, how potential secondary effects (e.g. second site T-DNA insertions, mosiacism, off-target gene editing) were examined.*

Flow Cytometry

Plots

Confirm that:

☐ The axis labels state the marker and fluorochrome used (e.g. CD4-FITC).

☒ The axis scales are clearly visible. Include numbers along axes only for bottom left plot of group (a 'group' is an analysis of identical markers).

☒ All plots are contour plots with outliers or pseudocolor plots.

☒ A numerical value for number of cells or percentage (with statistics) is provided.

Methodology

Sample preparation	Peripheral blood: Mouse blood was isolated from sacrificed mice from the vena cava following culling via cervical dislocation under isoflurane anaesthesia or sampled from live mice via the tail vein prior to MI. Red blood cells with lysed with 1× red blood cell lysis buffer (BioLegend) after which point samples were processed for flow cytometric analysis (See "Flow cytometry analysis of murine tissue" section).<br>Heart: Mice were sacrificed using cervical dislocation under isofluorane anaesthesia. Hearts were perfused via the left ventricle with cold PBS and excised before being dissected and enzymatically digested (in RPMI 1640 media; 450 U/ ml collagenase I, 125 U/ml collagenase XI, 60 U/ml DNase I, and 60 U/ml hyaluronidase, 30 minutes, 37oC degrees). Following digestion, mechanical dissociation was carried out (gentleMACS™) and cells were filtered through a 40-µm nylon mesh to obtain a single cell suspension.<br>Bone marrow: Femurs were isolated from sacrificed mice. Following removal of the epiphyses, the bone marrow was flushed with cold buffer of 5% FBS, 1mM EDTA (in DPBS). The cell suspension was filtered through a 40-µm nylon mesh after which it was processed for antibody staining and flow cytometry.
Instrument	LSRFortessaTM X-20 (BD), Aria Fusion (BD)
Software	Flowjo v10
Cell population abundance	For cardiac CD45+ cell sorting , purity was determined with re-analysis of sorted populations to determine CD45+ cell purity.
Gating strategy	Blood: Immune cell subsets were identified as follows: Monocytes (CD45high, CD11bhigh, Ly6Glow, CD115high, CX3CR1high, Ly6Clow/high), Neutrophils (CD45high, CD11bhigh, Ly6Ghigh), T-cells (CD45high CD11blow, Cd90high) and B-cells (CD45high, CD11blow, CD19high).<br>Heart: Cardiac immune cells were defined as: Monocytes (CD45high, Ly6Glow, Ly6Chigh, F4/80low), Macrophages (CD45high, Ly6Glow, Ly6Clow, F4/80high), Recruited Macrophages (CD45high, Ly6Glow, Ly6Clow, F4/80high, CCR2+), Neutrophils (CD45high, CD11bhigh, Ly6Ghigh), T-cells (CD45high CD11blow, CD90high) and B-cells (CD45high, CD11blow, CD19high).<br>Bone marrow:Bone marrow progenitor populations were defined as: LKs (Lineage-, c-kit+, Sca1-), LSKs (Lineage-, c-kit+, Sca1 +), GMPs (Lineage-, c-kit+, Sca1-, CD34+, CD16/32+), LT-HSCs (Lineage-, c-kit+, Sca1+, Flt3-, CD48-, CD150+).

☒ Tick this box to confirm that a figure exemplifying the gating strategy is provided in the Supplementary Information.
